# Two CYP82D Enzymes Function as Flavone Hydroxylases in the Biosynthesis of Root-Specific 4′-Deoxyflavones in *Scutellaria baicalensis*

**DOI:** 10.1016/j.molp.2017.08.009

**Published:** 2018-01-08

**Authors:** Qing Zhao, Meng-Ying Cui, Olesya Levsh, Dongfeng Yang, Jie Liu, Jie Li, Lionel Hill, Lei Yang, Yonghong Hu, Jing-Ke Weng, Xiao-Ya Chen, Cathie Martin

**Affiliations:** 1 Shanghai Key Laboratory of Plant Functional Genomics and Resources, Shanghai Chenshan Botanical Garden, Shanghai Chenshan Plant Science Research Center, Chinese Academy of Sciences, Shanghai, China; 2 Department of Metabolic Biology, John Innes Centre, Norwich NR4 7UH, UK; 3 Whitehead Institute for Biomedical Research, 455 Main Street, Cambridge, MA 02142, USA; 4 Department of Biology, Massachusetts Institute of Technology, Cambridge, MA 02139, USA; 5 College of Life Sciences, Zhejiang Sci-Tech University, Key Laboratory of Plant Secondary Metabolism and Regulation of Zhejiang Province, Hangzhou 310018, China; 6 State Key Laboratory of Plant Molecular Genetics, CAS Center for Excellence in Molecular Plant Sciences, Shanghai Institute of Plant Physiology and Ecology, Chinese Academy of Sciences, Shanghai, China

**Keywords:** *Scutellaria baicalensis*, Huangqin, baicalein, wogonin, flavone 6-hydroxylase, flavone 8-hydroxylase

## Abstract

Baicalein, wogonin, and their glycosides are major bioactive compounds found in the medicinal plant *Scutellaria baicalensis* Georgi. These flavones can induce apoptosis in a variety of cancer cell lines but have no effect on normal cells. Furthermore, they have many additional benefits for human health, such as anti-oxidant, antiviral, and liver-protective properties. Here, we report the isolation and characterization of two CYP450 enzymes, SbCYP82D1.1 and SbCYP82D2, which function as the flavone 6-hydroxylase (F6H) and flavone 8-hydroxylase (F8H), respectively, in *S. baicalensis*. SbCYP82D1.1 has broad substrate specificity for flavones such as chrysin and apigenin and is responsible for biosynthesis of baicalein and scutellarein in roots and aerial parts of *S. baicalensis*, respectively. When the expression of *SbCYP82D1.1* is knocked down, baicalin and baicalein levels are reduced significantly while chrysin glycosides accumulate in hairy roots. SbCYP82D2 is an F8H with high substrate specificity, accepting only chrysin as its substrate to produce norwogonin, although minor 6-hydroxylation activity can also be detected. Phylogenetic analysis suggested that SbCYP82D2 might have evolved from SbCYP82D1.1 via gene duplication followed by neofunctionalization, whereby the ancestral F6H activity is partially retained in the derived SbCYP82D2.

## Teaser

We report the characterization of two CYP450 enzymes, which 6- and 8-hydroxylate chrysin to form the 4′-deoxyflavone bioactives in roots of *Scutellaria baicalensis*. Like the main 4′-deoxyflavone enzymes, these decorating enzymes have evolved their functionalities by convergence with the more ubiquitous 4′-hydroxyflavone pathway enzymes.

## Introduction

*Scutellaria baicalensis* Georgi or Huangqin is a medicinal plant used widely in China and many other Asian countries. Its dried roots have been used for treating lung infections, liver problems, inflammation, diarrhea, and dysentery for thousands of years (Shang et al., 2010, Zhao et al., 2016a). The pharmacological activities of *S. baicalensis* have been attributed mainly to the large amounts of 4′-deoxyflavones, which accumulate specifically in roots (root-specific flavones [RSFs]): baicalin, wogonoside, and their aglycones, baicalein and wogonin (for structures, see Supplemental Table 1) (Makino et al., 2008, Li-Weber, 2009). These flavones are reported to have various benefits for human health, such as anti-fibrotic activity in the liver and anti-cancer properties (Gao et al., 2011, Yang et al., 2012). RSFs induce apoptosis specifically in cancer cells while having no effect on normal cells (Fox et al., 2012, Chen et al., 2013). It would be of interest to enhance the production of RSFs in *Scutellaria* plants or, alternatively, synthesize them in novel hosts (chassis). Elucidation of the biosynthetic pathways for the RSFs would lay a solid foundation for these applications.

**Figure 1:**
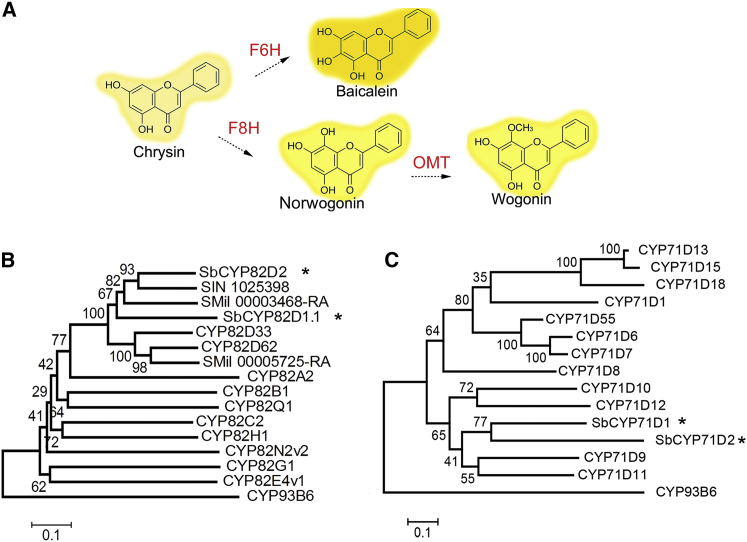
The Proposed Downstream Pathway for 4′-Deoxyflavones and Phylogenetic Analysis of CYP82Ds and CYP71Ds. **(A)** The proposed biosynthetic pathways for baibalein and wogonin from the flavone, chrysin. **(B)** Bootstrap consensus tree of CYP82D subfamily. The maximum-likelihood method was used to construct this tree with 1000 replicate bootstrap support. The tree was rooted with CYP93B6. GenBank IDs of the proteins used and their species names: CYP82D33, JX162212, *Ocimum basilicum*; CYP82D62, JX162214, *Mentha piperita*; CYP82A2, CAA71515, *Glycine max*; CYP82B1, AAC39454, *Eschscholzia californica*; CYP82Q1, ABB20912, *Stevia rebaudiana*; CYP82C2, O49394, *Arabidopsis thaliana*; CYP82H1, AAS90126, *Ammi majus*; CYP82N2v2, BAK20464, *Eschscholzia californica*; CYP82G1, NP189154, *Arabidopsis thaliana*; CYP82E4v1, ABA07805, *Nicotiana tabacum*; CYP93B6, BAB59004.1, *Perilla frutescens*. SIN1025398, SMil00003468-RA, and Smil00005725-RA are protein locus from *Salvia miltiorrhiza* genome sequencing database. Proteins from *Scutellaria baicalensis* studied in this work are marked with asterisks. **(C)** Bootstrap consensus tree of CYP71D subfamily. The maximum-likelihood method was used to construct this tree with 1000 replicate bootstrap support. The tree was rooted with CYP93B6. GenBank IDs of the proteins used and their species names: CYP71D13, Q9XHE7.1, *Mentha piperita*; CYP71D15, Q9XHE6.1, *Mentha piperita*; CYP71D18, Q6WKZ1.1, *Mentha piperita*; CYP71D1, ACD42776.1, *Catharanthus roseus*; CYP71D55, A6YIH8.1, *Hyoscyamus muticus*; CYP71D6, P93530.1, *Solanum chacoense*; CYP71D7, P93531.1, *Solanum chacoense*; CYP71D8, O81974.1, *Glycine max*; CYP71D10, NP_001236165.1, *Glycine max*; CYP71D12, P98183.1, *Catharanthus roseus*; CYP71D9, NP_001304582.1, *Glycine max*; CYP71D11, O22307.1, *Lotus japonicus*. Proteins from *S. baicalensis* studied in this work are marked with an asterisk. For amino acid sequence alignments, see Supplemental Figure 10.

*Scutellaria* RSFs are not produced from naringenin, the common intermediate for most flavones, but from pinocembrin, a 4′-deoxyflavanone (Supplemental Table 1). We have shown that an alternative flavone pathway has evolved in *S. baicalensis*, which has recruited a specific cinnamoyl-coenzyme A (CoA) ligase (SbCLL-7) to form cinnamoyl-CoA. Cinnamoyl-CoA is condensed with malonyl-CoA by a specific isoform of chalcone synthase, SbCHS-2, and isomerized by chalcone isomerase (SbCHI) to form pinocembrin. A newly evolved isoform of flavone synthase II (FNSII), SbFNSII-2, which accepts only pinocembrin as a substrate, then converts pinocembrin to chrysin (Supplemental Table 1), the primary flavone without a 4′-OH group (Zhao et al., 2016b). Chrysin may subsequently be decorated by flavonoid 6- or 8-hydroxylases, 8-*O*-methyltransferases (Figure 1A), and glycosyltransferases to produce a range of RSFs in *S. baicalensis* (Zhao et al., 2016a). Neither the 6- nor the 8-hydroxylase enzymes nor the genes encoding them have been identified in this medicinal plant.

Two CYP450 family enzyme activities that might produce 6-OH flavones have been reported. Flavonoid 6-hydroxylase, or CYP71D9, was first reported from soybean, where it converts the flavanone liquiritigenin to 4′,6,7-trihydrosyflavanone. This enzyme could efficiently catalyze the hydroxylation of other flavanones including eriodictyol and naringenin as well as the dihydroflavonol, dihydrokaempferol. However, it has low activity toward flavones (Latunde-Dada et al., 2001). In 2013, Berim and Gang reported two flavone 6-hydroxylases (F6Hs), one from sweet basil and the other from mint, named CYP82D33 and CYP82D62, respectively. The enzymes could convert the flavone genkwanin to produce 7-methylscutellarein. However, they could hydroxylate only the C-6 of 7-methylated flavones, like genkwanin, and had very low activity on flavones lacking a 7-methyl group, such as apigenin. Extended incubation time and excess protein were required to produce very small amount of scutellarein when apigenin was used as a substrate. Consequently, the authors proposed that 7-methylation is a prerequisite for 6-hydroxylation by CYP82D33 and CYP82D62 (Berim and Gang, 2013).

Flavone 8-hydroxylase (F8H) activity was first detected in microsomes extracted from *Chrysanthemum segetum* petals. This activity was detected in the presence of NADPH and FAD and the enzyme could 8-hydroxylate both flavonols and flavones, but the protein had no activity on dihydroflavonol, glycosylated flavonols, or glycosylated flavones (Halbwirth and Stich, 2006). Additionally, in 2014 Berim and colleagues isolated an 8-hydroxylase from sweet basil that could convert salvigenin or crisimaritin to 8-hydroxysalvigenin or 8-hydroxycrisimaritin. This plastid-localized enzyme belongs to the protein family of Rieske-type oxygenases and is highly expressed in the trichomes of sweet basil leaves (Berim et al., 2014).

Although CYP71D9 from soybean could 6-hydroxylate both flavanones and flavones, there is no experimental evidence showing that its homolog in plants of the family Lamiaceae might be responsible for 6-hydroxylation of RSFs. Both sweet basil and *Scutellaria* belong to the Lamiaceae family, and might have genes encoding proteins from the same subfamily that perform similar enzymatic reactions to hydroxylate the 6-position of flavonoids. However, the 4′-deoxyflavone biosynthetic pathway appears to have evolved relatively recently, and the genes encoding enzymes involved in the biosynthesis of baicalein probably evolved independently of those involved in the synthesis of 7-*O*-methylflavones. Furthermore, the F8H of sweet basil is localized in plastids (Berim et al., 2014), presenting a logistical problem for the decoration of 4′-deoxyflavones such as norwogonin, because the previous biosynthetic step involving the enzyme SbFNSII-2/CYP93B25 is ER-localized. Consequently, more research is needed to uncover which enzyme is responsible for the 8-hydroxylation of flavones in the wogonin biosynthetic pathway.

Here, we report the identification and characterization of two CYP82D enzymes from *S. baicalensis*. SbCYP82D1.1 encodes an F6H with broad specificity for flavones, and is responsible for the biosynthesis of baicalein and scutellarein. This enzyme can convert flavones without 7-*O*-methyl groups such as chrysin and apigenin at high efficiency and is different from CYP82D33 and CYP82D62, which have moderate activity on 7-*O*-methylflavones. *SbCYP82D2* likely evolved from *SbCYP82D1.1* through an ancestral gene duplication event. Interestingly, the protein encoded by *SbCYP82D2* is an F8H, and accepts only chrysin as substrate to produce norwogonin, which is the precursor of wogonin. Our present study extends our previous work to elucidate the entire biosynthetic pathway for baicalein and an additional hydroxylation step in the pathway to produce wogonin.

## Results

### Identification of cDNAs Encoding Putative Flavone 6-Hydroxylases in *S. baicalensis*

Two types of CYP450 proteins that can 6-hydroxylate flavonoids have been reported. They are CYP71D9 from soybean that converts the flavanone liquiritigenin to 4′,6,7-trihydroxyflavanone (Latunde-Dada et al., 2001), and CYP82D33 from sweet basil or CYP82D62 from mint that produces 7-methylscutellarein from genkwanin (Berim and Gang, 2013). To identify genes that may hydroxylate chrysin in *Scutellaria* and thus might be involved in baicalein biosynthesis, we screened for contigs from RNA-sequencing (RNA-seq) databases of hairy roots and flowers (Zhao et al., 2016b) encoding proteins that were annotated as CYP71D or CYP82D, and performed BLAST searches using CYP71D9 and CYP82D33 as bait. This work identified two cDNAs encoding putative CYP71D proteins and two encoding CYP82D proteins, which we named *SbCYP71D1*, *SbCYP71D2*, *SbCYP82D1*, and *SbCYP82D2*, respectively (Figure 1B and 1C). In RNA-seq studies, fragments per kilobase of transcript per million mapped reads (FPKM) showed the abundance of the transcripts of these genes (Supplemental Table 2). The relative transcript levels of *SbCYP71D1* were quite low in both hairy root and flower tissues, at 3.464 and 1.9377, respectively, compared with the levels of *SbCYP71D2*, which were 127.4283 and 8.5701, respectively. *SbCYP82D1* had FPKM 59.7978 and 10.7494 in hairy roots and flower samples, respectively. *SbCYP82D2* also had high relative transcript levels in hairy roots with FPKM 195.0871, while the levels in flowers were much lower at 3.3727.

Based on the sequences from the RNA-seq databases, we successfully obtained the open reading frames (ORFs) of *SbCYP71D1*, *SbCYP71D2*, and *SbCYP82D2* by RT–PCR. The ORFs of *SbCYP71D1* and *SbCYP71D2* were 1506 bp and 1530 bp long, encoding proteins of 501 amino acids (aa) and 509 aa, respectively. Two cDNAs were isolated using primer pairs based on the *SbCYP82D1* sequence (Supplemental Table 3), which may be the result of alternative splicing. We named them as *SbCYP82D1.1* and *SbCYP82D1.2. SbCYP82D1.1* is 72 bp longer than the 1482-bp CDS (coding sequence) of *SbCYP82D1.2* and encodes a protein 24 aa longer (517 aa) than the SbCYP82D1.2 protein of 493 aa. The 1581-bp coding sequence of *SbCYP82D2* encoded a 526-aa protein. SbCYP82D1.1 was 64% identical to SbCYP82D2 at the amino acid level, and SbCYP82D1.1 and SbCYP82D2 had 60% and 67% identity to CYP82D33 from sweet basil, respectively.

### Screening of Flavone 6-Hydroxylases Using an *In Vivo* Assay in Yeast

**Figure 2:**
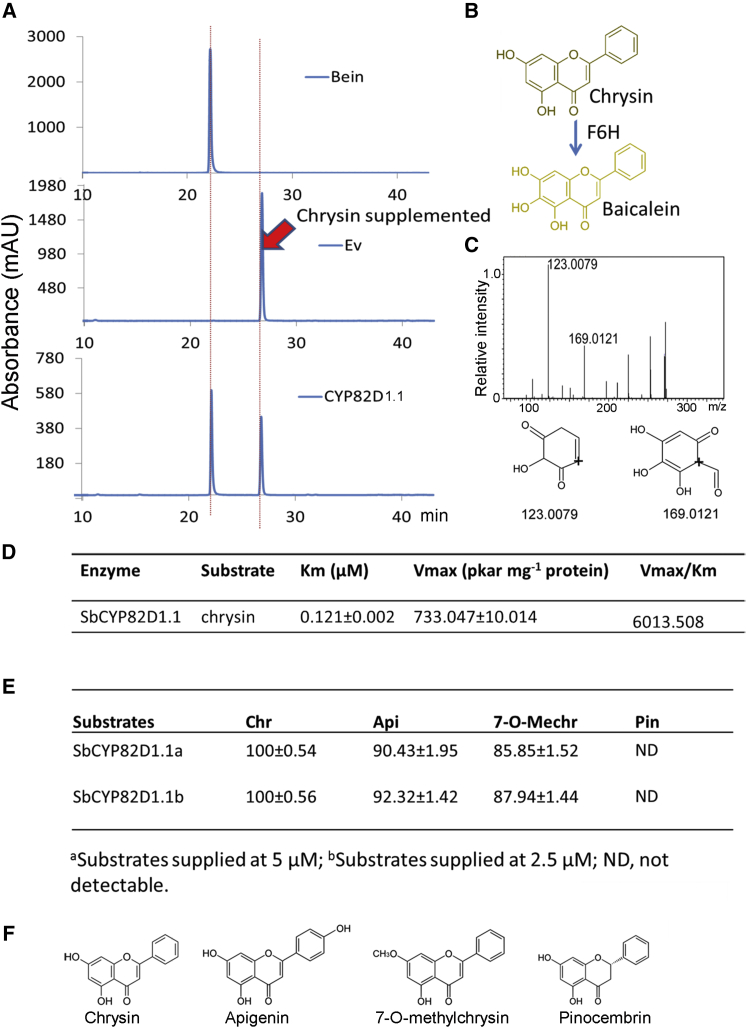
Characterization of SbCYP82D1.1 Enzyme Activity. **(A)** Assays of activity in yeast *in vivo*. HPLC analysis of yeast samples incubated with chrysin: top, baicalein standard; middle, yeast with empty vector (Ev); bottom, yeast expressing SbCYP82D1.1, where a new peak with the same retention time as baicalein was found. Bein, baicalein. **(B)** The proposed reaction catalyzed by SbCYP82D1.1. **(C)** MS2 and fragmentation patterns of the new compound produced by SbCYP82D1.1 when expressed in yeast, which was identical to baicalein. **(D)** Kinetic analyses of CYP82D1.1 determined *in vitro* following expression in yeast; each dataset represents the mean ± SE from triplicate measurements. **(E)** Relative turnover rate of CYP82D1.1 with apigenin, 7-*O*-methylchrisin, or pinocembrin used as substrates. Chr, chrysin; Api, apigenin; 7-*O*-Mechr, 7-*O*-methylchrysin; Pin, pinocembrin. ND, not detected; each dataset represents the mean ± SE from triplicate measurements, for 5 μM chr, 100% = 707.138 pkat mg^−^^1^ protein; for 2.5 μM chr, 100% = 702.098 pkat mg^−^^1^ protein. **(F)** Structures of the substrates used in the CYP82D1.1 *in vitro* assays.

To identify the enzymatic activities of the isolated CYP450 genes rapidly, we undertook an *in vivo* yeast assay on strains expressing each CYP450 in *Saccharomyces cerevisiae* WAT11, an engineered strain overexpressing an *Arabidopsis* NADPH-cytochrome P450 reductase gene (Pompon et al., 1996). The putative substrate, chrysin, was added to the medium of WAT11 strains carrying an empty vector, or expressing *SbCYP71D1*, *SbCYP71D2*, *SbCYP82D1.1*, *SbCYP82D1.2*, and *SbCYP82D2*, and incubated overnight. The yeast cells were harvested, extracted, and analyzed by liquid chromatography–mass spectrometry (LC–MS). We did not observe any substantial new products in the strains expressing SbCYP71D1, SbCYP71D2, SbCYP82D1.2, or SbCYP82D2 compared with the empty vector control, but a large, new peak was detected in extracts from yeast expressing SbCYP82D1.1 (Figure 2A). This new product had a retention time identical to that of the authentic baicalein standard, and the strain converted more than half of the chrysin substrate added (Figure 2A). Furthermore, the peak had the same tandem MS (MS/MS) pattern as the baicalein standard, with fragments of *m*/*z* 123.0079 and 169.0121. We therefore identified this product as baicalein and the CYP82D1.1 enzyme as a flavone 6-hydroxylase (Figure 2A–2C).

### *In Vitro* Enzyme Assays and Kinetic Study of CYP82D1.1

Microsomal proteins were extracted from the WAT11 strains expressing SbCYP82D1.1 and were assayed against chrysin. In accordance with the results *in vivo*, the enzyme produced baicalein after incubation with NADPH (Supplemental Figure 1). Kinetic parameters were determined for chrysin under initial rate conditions. SbCYP82D1.1 converted chrysin at high efficiency, with an apparent *K*_M_ value of 0.121 μM and apparent maximal velocity value of 733.047 pkat mg^−^^1^ protein (Figure 2D). The activities of the enzyme were also assayed with three other flavonoids possessing structures similar to chrysin, namely pinocembrin, apigenin, and 7-*O*-methylchrysin. The results showed that SbCYP82D1.1 was promiscuous, and could use any flavone as a substrate to produce its 6-hydroxylated form with comparable efficiency (Figure 2E and Supplemental Figure 2). This indicated that the lack of a 4′-OH or the presence of a 7-*O*-methyl group did not preclude the activity of SbCYP82D1.1. However, this enzyme could not hydroxylate the flavanone pinocembrin, showing that the two to three double bonds in flavones are indispensable for this reaction (Figure 2E and 2F; Supplemental Figure 2).

### Expression Profile of *SbCYP82D1.1* and Effects of Its Silencing on 4′-Deoxyflavone Synthesis

**Figure 3:**
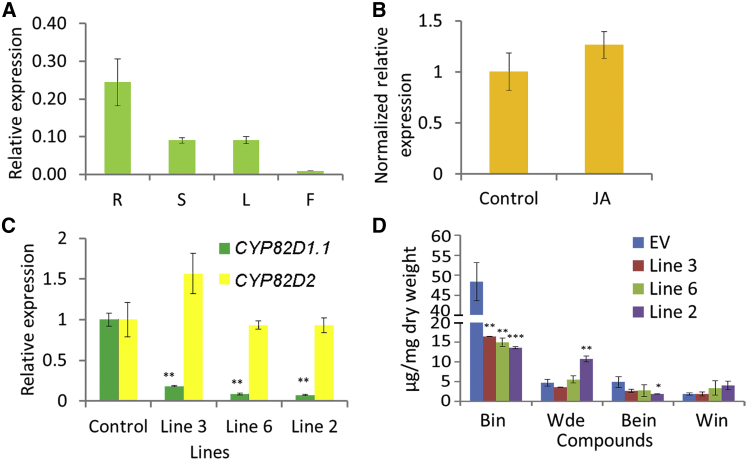
Expression Patterns of *SbCYP82D1.1* and the Effects of Silencing *SbCYP82D1.1* by RNAi. **(A)** Relative levels of *SbCYP82D1.1* transcripts compared with β-actin were determined by qRT–PCR analyses performed on total RNA extracted from different organs of *S. baicalensis*. R, roots; S, stems; L, leaves; F, flowers. **(B)** Relative expression of *SbCYP82D1.1* after control and MeJA treatment for 24 h. The expression levels were normalized to corresponding values from mock treatments. **(C)** Relative transcript levels of *SbCYP82D1.1* in different RNAi-silenced hairy root lines measured by qRT–PCR. **(D)** Measurements of RSFs from the *SbCYP82D1.1* selected RNAi lines used for transcript analysis. Bin, baicalin; Wde, wogonoside; Bein, baicalein; Win, wogonin. SEs were calculated from three biological replicates. *P < 0.05, **P < 0.01, and ***P < 0.001 (Student's *t*-test).

The transcript levels of *SbCYP82D1.1* were compared in four different organs of *S. baicalensis* by qRT–PCR. In accordance with the RNA-seq data, expression of *SbCYP82D1.1* was highest in roots (Figure 3A). The relative expression in roots was 0.224, followed by stems at 0.090 and leaves at 0.091, with the lowest transcript levels in flowers. The gene was not significantly induced by methyl jasmonate (Figure 3B), although methyl jasmonate induced accumulation of baicalein, wogonin, and their glycosides in hairy roots. These results suggest that genes downstream in RSF biosynthesis are regulated differently to the upstream genes such as *SbCHS*-*2* and *SbFNSII*-*2*, which are induced substantially by methyl jasmonate (Zhao et al., 2016b). The strong expression of *SbCYP82D1.1* in roots may account for the large amounts of baicalein and baicalin in the roots of *S. baicalensis*. However, since SbCYP82D1.1 can also use apigenin as a substrate and has about 40% expression level in stems and leaves compared with roots, this enzyme might also be responsible for production of scutellarein and scutellarin in the aerial parts of *S. baicalensis* plants (Islam et al., 2011, Zhao et al., 2016b).

Hairy root-mediated RNAi was employed to confirm the role of *CYP82D1.1* in the RSF biosynthetic pathway. We screened three different hairy root lines with different degrees of downregulation of transcript levels of this gene. qRT–PCR showed reduced expression levels of *CYP82D1.1* in line 3, line 6, and line 2 with residual transcript levels of 18%, 8.4%, and 7.3%, respectively, compared with controls (Figure 3C). Baicalin was significantly reduced in the three RNAi hairy root lines, with 33.3%, 31.2%, and 27% of the baicalin levels of controls, respectively (Figure 3D and Supplemental Figure 3). Significant reductions in baicalein were detected only between line 2 and the empty vector control. Wogonoside was increased to 10.73 mg g^−^^1^ dry weight (DW) in line 2 compared with 4.7 mg g^−^^1^ DW in controls. Wogonin levels were also increased slightly in lines 2 and 6, although these changes were not statistically significant. A new peak, detected in *CYP82D1.1* RNAi lines, with an *m*/*z* of 431, was identical to chrysin glucuronide (Supplemental Figure 3). MS/MS data showed a fragment of *m*/*z* 255.2, identical to chrysin, the substrate of CYP82D1.1. These results offered direct evidence that CYP82D1.1 is involved in baicalein and baicalin biosynthesis in *S. baicalensis* and functions as a flavone 6-hydroxylase.

### A Putative Rieske-type Oxygenase Is Not Responsible for 8-Hydroxylation of Chrysin in *S. baicalensis*

**Figure 4:**
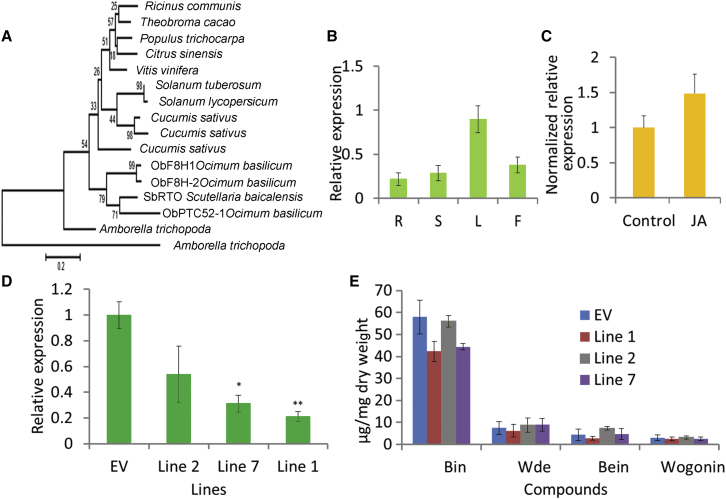
Expression Patterns of *SbRTO* and the Effects of Silencing *SbRTO* by RNAi. **(A)** Bootstrap consensus tree of RTO family. The maximum-likelihood method was used to construct this tree with 1000 replicate bootstrap support. Species names and GenBank IDs for peptide sequences used were: *Ricinus communis*, EEF2899; *Theobroma cacao*, EOY32295; *Populus trichocarpa*, EEE83915; *Citrus sinensis*, XP006488912; *Vitis vinifera*, XP002283592; *Solanum tuberosum*, XP006364436; *Solanum lycopersicum*, XP004237332; *Cucumis sativus*, XP004144089, XP004144087, XP004144144; *Amborella trichopoda*, ERN20453, ERN07127; *Ocimum basilicum*, AII16849 (ObF8H-1), AII16848 (ObF8H-2), AII16851(ObPTC52-1). For amino acid sequence alignments, see Supplemental Figure 10. **(B)** Relative levels of *SbRTO* transcripts compared with β-actin determined by qRT–PCR analyses of cDNA from total RNA extracted from different *S. baicalensis* organs. R, roots; S, stems; L, leaves; F, flowers. **(C)** Relative expression of *SbRTO* following MeJA treatment for 24 h. The expression levels were normalized to corresponding values from mock treatments. **(D)** Silencing of *SbRTO* in different RNAi hairy root lines was measured by monitoring relative transcript levels by qRT–PCR. **(E)** Measurements of RSFs from the *SbRTO*-RNAi lines used for transcript analyses. Bin, baicalin; Wde, wogonoside; Bein, baicalein; Win, wogonin. SEs were calculated from three biological replicates. *P < 0.05 and **P < 0.01 (Student's *t*-test).

A Rieske-type oxygenase, ObF8H from sweet basil (*Ocimum basilicum*), has been reported, which can 8-hydroxylate the flavones salvigenin or crisimaritin to produce 8-hydroxysalvigenin or 8-hydroxycrisimaritin (Berim et al., 2014). We speculated that the homolog of ObF8H in *Scutellaria* might also play a similar role in the synthesis of wogonin and wogonoside. We undertook a BLAST search using the sequence of ObF8H and found a unigene with an ORF of 1572 bp in our transcriptome databases (Figure 4A). The full-length ORF of this gene was isolated using RT–PCR, and we named it *SbRTO* (Rieske-type oxygenase). However, this gene had highest expression levels in leaves, as shown by qRT–PCR, with 1.37-, 2.14-, and 3.12-fold higher transcript levels than in flowers, stems, and roots, respectively (Figure 4B). This gene was not induced by MeJA (Figure 4C). Consequently, the expression patterns for *SbRTO* were not aligned with RSF accumulation and were different from the expression patterns of the genes we had identified earlier that were involved in the RSF pathway, such as *SbCLL*-*7*, *SbCHS*-*2*, and *SbFNSII*-*2*, which all showed highest expression in roots. To determine whether SbRTO could function as a F8H, we expressed the protein in yeast, fed the strain with chrysin, and grew it overnight. However, no new product was detected from the extract of the yeast expressing SbRTO and fed chrysin, compared with extracts of empty vector controls (Supplemental Figure 4).

RNAi technology was used to investigate whether *SbRTO* was involved in RSF biosynthesis. Three independent hairy root lines that had been transformed with the SbRTO-RNAi T-DNA as indicated by dsRed fluorescence were analyzed. Transcripts of the target *SbRTO* gene were significantly reduced in two of the lines, with 31.1% and 20.9% of the transcript levels of controls in line 7 and line 1, respectively (Figure 4D). However, silencing this gene had no effect on the levels of any of the four RSFs (Figure 4E). Together, these results established that SbRTO is not responsible for 8-hydroxylation of chrysin and is not involved in the synthesis of wogonin and wogonoside. Our results suggest that different genes encoding enzyme(s) from those in sweet basil evolved in *Scutellaria* to carry out this function.

### Identification of Genes Encoding Flavone 8-Hydroxylase Activity in *S. baicalensis*

**Figure 5:**
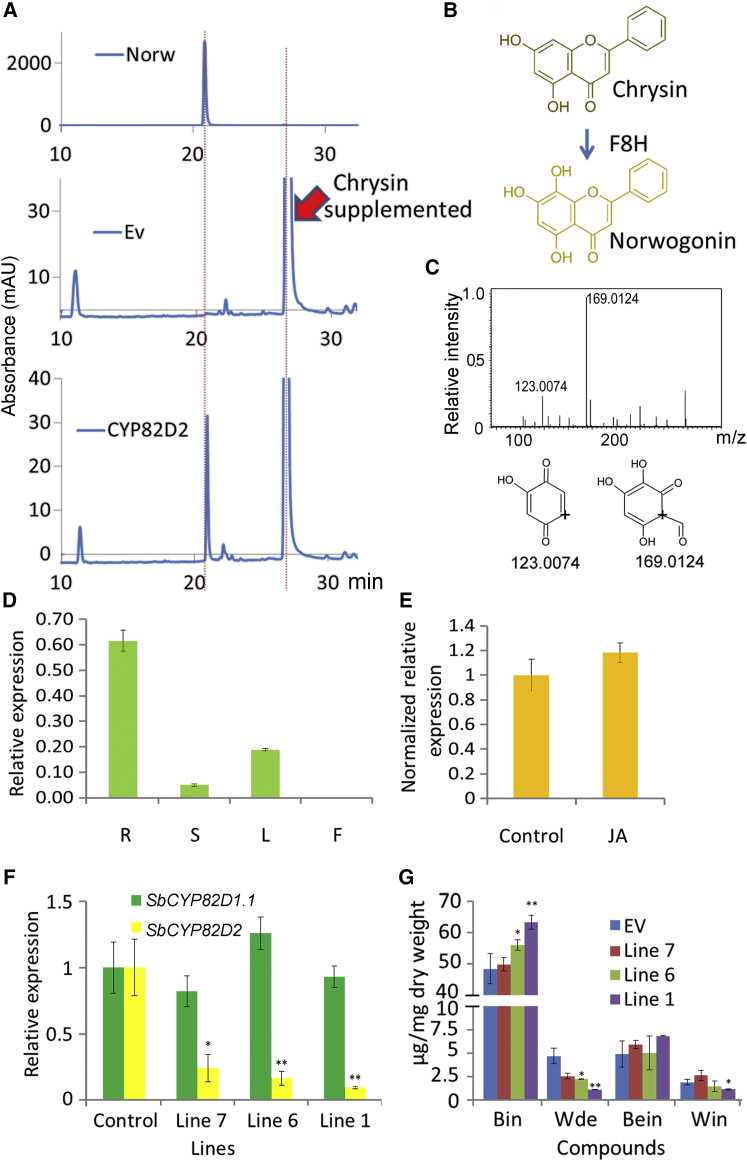
Characterization of SbCYP82D2 Enzyme Activity, Gene Expression Analysis, and RNAi of *SbCYP82D2* in Hairy Roots. **(A)** HPLC analysis of yeast samples incubated with chrysin *in vivo*. Top, norwogonin standard; middle, yeast carrying empty vector; bottom, yeast expressing SbCYP82D2, where a new peak with the same retention time as norwogonin was found. Norw, norwogonin. **(B)** The proposed reaction catalyzed by SbCYP82D2. **(C)** MS2 and fragmentation patterns of the new compound produced by SbCYP82D2 expressed in yeast. The fragmentation patterns were identical to those for norwogonin. **(D)** Relative levels of *SbCYP82D2* transcripts compared with β-actin determined by qRT–PCR analyses performed on cDNA from total RNA extracted from different organs. R, roots; S, stems; L, leaves; F, flowers. **(E)** Relative expression of *SbCYP82D2* following MeJA treatment for 24 h. The expression levels were normalized to corresponding values from mock treatments. **(F)** Silencing of *SbCYP82D2* in RNAi hairy roots was measured by monitoring relative transcript levels by qRT–PCR. **(G)** Measurements of RSFs from the *SbCYP82D2* RNAi lines used for transcript analysis. Bin, baicalin; Wde, wogonoside; Bein, baicalein; Win, wogonin. SEs were calculated from three biological replicates. *P < 0.05 and **P < 0.01 (Student's *t*-test).

We then recalled that a small peak, possibly equivalent to norwogonin, had been observed in SbCYP82D2-expressing WAT11 yeast fed chrysin during the screen for flavone 6-hydroxylase activity. We repeated this experiment and allowed the fermentation to proceed for longer (from 16 h to 24 h) to enhance product formation. Interestingly, the product of SbCYP82D2 ran about 1 min before the baicalein standard on our high-performance liquid chromatography (HPLC) system and had the same retention time as the norwogonin standard, which is the 8-hydroxylated chrysin and the precursor of wogonin (Figure 5A). We analyzed the yeast extracts on quadrupole time-of-flight (Q-TOF), and MS/MS data showed that the product of SbCYP82D2 had the same fragmentation pattern as authentic norwogonin, with fragments of *m*/*z* 123.0074 and 169.0124. The abundance of the 169.0124 fragment was about 3-fold higher than the 123.0074 fragment (Figure 5C). Although baicalein also has MS/MS fragments of *m*/*z* 123.0079 and 169.0121, the abundance of the former is about 2-fold higher than that of the latter and is clearly distinct from norwogonin.

To confirm this result, we extracted microsomal proteins from the yeast strain expressing SbCYP82D2 and carried out an *in vitro* enzyme assay using chrysin as the substrate. In accordance with previous results, this enzyme could convert chrysin to norwogonin in the presence of NADPH with an apparent *K*_M_ of 1.051 μM and *V*_max_ of 37.453 pkat mg^−^^1^ protein (Supplemental Figure 5). The activity of SbCYP82D2 was also assayed with pinocembrin, apigenin, and 7-*O*-methylchrysin, but no new peaks were found, showing this enzyme to be very specific for chrysin with no activity on related flavones or flavanones (Supplemental Figure 6).

### Expression Pattern of *SbCYP82D2* and the Effect of Its Silencing in Hairy Roots of *S. baicalensis*

FPKM values from RNA-seq showed that the transcript levels of *SbCYP82D2* in hairy roots were 58-fold higher than those in flowers, indicating that *SbCYP82D2* might be expressed relatively specifically in roots. The expression pattern of *SbCYP82D2* was confirmed further by qRT–PCR, which showed that this gene had its highest transcript levels in roots, where expression relative to actin was 0.62, followed by leaves and stems, with relative expression values of 0.19 and 0.05, respectively (Figure 5D). Very few transcripts of *SbCYP82D2* were detected in flowers. Like *SbCYP82D1.1*, *SbCYP82D2* was not induced by MeJA (Figure 5E).

RNAi was employed to confirm the role of *SbCYP82D2* in the RSF pathway. Transcript levels of *SbCYP82D2* were reduced to 24%, 16%, and 9% in line 7, line 6, and line 1, respectively, compared with levels in empty vector controls (Figure 5F). Accordingly, levels of wogonoside were decreased from 4.70 mg g^−^^1^ DW in the empty vector control to 2.22 and 1.12 mg g^−^^1^ DW in line 6 and line 1, respectively (Figure 5G and Supplemental Figure 7). Wogonin was also reduced to 1.12 mg g^−^^1^ DW in line 1 compared with 1.90 mg g^−^^1^ DW in empty vector (Figure 5G). Interestingly, baicalin levels were increased slightly in line 6 and line 1. No significant differences in baicalein levels were observed in any of the RNAi lines (Figure 5G).

### Phylogenetic Analysis of SbCYP82Ds

The two SbCYP82Ds function as flavone 6- and 8-hydroxylases in *S. baicalensis*, and belong to the same subfamily of CYP450 proteins. A phylogenetic tree was constructed to evaluate the evolutionary relationships between these proteins and with other CYP82D proteins. Both SbCYP82D1 and SbCYP82D2 were grouped in a clade with proteins from other plants in the family of Lamiaceae (Figure 1B and 1C), which are clearly separated from soybean CYP82A2, a protein with unknown function induced by fungal elicitor (Schopfer and Ebel, 1998). The Lamiaceae branch of the CYP82D family tree also includes CYP82D33 from sweet basil, CYP82D62 from mint, and three proteins from salvia that recently had its genome sequenced (SIN 1025389, SMil 00003468, and SMil 00005725) (Xu et al., 2016). This tree implies that SbCYP82D2 may have evolved from SbCYP82D1 before emergence of *Scutellaria*, since two proteins from salvia with unknown functions (which do not produce 4′-deoxyflavones) also diverged from the ancestor of SbCYP82D1. These proteins branched into two paths, suggesting that the gene encoding SbCYP82D1.1 underwent neofunctionalization from the ancestor of *CYP82D33* or *CYP82D62* in the Lamiaceae, since CYP82D33 or CYP82D62 have activity only on 7-*O*-methylflavones. The gene encoding SbCYP82D2 may also have undergone neofunctionalization from the gene encoding SbCYP82D1.1.

Since our phylogenetic analysis suggested that SbCYP82D2 might have evolved from SbCYP82D1, we hypothesized that this enzyme may have retained some residual activity of its ancestral progenitor. We analyzed extracts of the yeast *in vivo* assays using Q-TOF to examine compounds with *m*/*z* 271, because baicalein and norwogonin have the same molecular mass. We traced these fragments and found that *m*/*z* 271 formed two peaks which ran at 5.703 min and 5.936 min, respectively, on our system (Supplemental Figure 8). The faster peak had a larger fragment of *m*/*z* 169.0106, characteristic of norwogonin, while the slower peak had a larger fragment of *m*/*z* 123.0053, identical to the baicalein standard. These results confirmed that SbCYP82D2 could make tiny amounts of baicalein, although its main product was norwogonin.

### Modeling of CYP82D Protein Structures in Relation to Their Substrate Preferences

**Figure 6:**
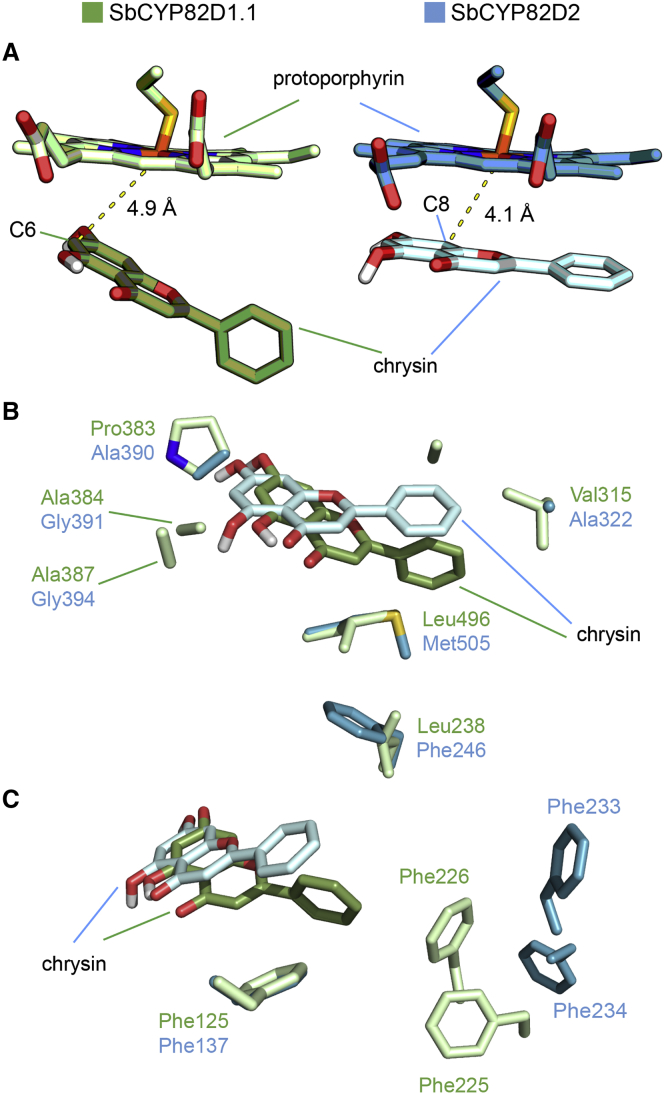
Structural Modeling of Chrysin Binding with SbCYP82D1.1 and SbCYP82D2. **(A)** Ligand modeling results indicate that chrysin binds in different orientations in the SbCYP82D1.1 (green) and SbCYP82D2 (blue) binding sites. The tilted conformation of chrysin in the SbCYP82D1.1 active site causes the 6-carbon (C6) to be 4.9 Å away from the protoporphyrin iron, whereas the flat conformation in the SbCYP82D2 active site places the 8-carbon (C8) 4.1 Å away from the protoporphyrin iron. **(B)** Bulky substrate-proximal residues in SbCYP82D1.1 such as Pro383, Ala384, and Ala387 may shift chrysin binding, while additional bulky residues on the opposite end such as Val315, Leu496, and Leu238 may cause the substrate to tilt. **(C)** Phe125, Phe225, and Phe226 in SbCYP82D1.1 form a nearby hydrophobic pocket that may stabilize the tilted binding of chrysin. The corresponding residues in SbCYP82D2 are located too far away to mediate the same effect.

To determine the structural basis for F6H and F8H activity in each of the CYP450s, we modeled chrysin binding in CYP82D1.1 and CYP82D2 (Figure 6). We observed that chrysin bound in the same orientation in both of the enzyme active sites, but that it occupied a different, tilted position in CYP82D1.1 (Figure 6A). An investigation of the ligand-binding pocket revealed some substitutions between the two enzymes that may be responsible for the differences in binding modes. For instance, residues surrounding both ends of the substrate had bulkier side chains in CYP82D1.1 than in CYP82D2. Therefore, while residues such as Pro383, Ala384, and Ala387 pushed the substrate downward in the active site, residues on the other end such as Leu238, Val315, Ala319, and Leu496 caused the chrysin molecule to tilt (Figure 6B) in CYP82D1.1. Meanwhile, a hydrophobic pocket composed of surrounding phenylalanine residues, absent in CYP82D2, stabilized this tilted conformation (Figure 6C). As a result, the chrysin 6-carbon was within 4.9 Å of the protoporphyrin iron of the active site in CYP82D1.1, and the relative proximity of the 6-carbon compared with the 8-carbon to the hydroxylation site might drive the production of baicalein (Figure 6A). Similarly, the binding mode of chrysin in the CYP82D2 active site brought the chrysin 8-carbon within 4.1 Å of the protoporphyrin iron, resulting in closer proximity of the 8-carbon relative to the 6-carbon to the reaction center and thus promoted the formation of norwogonin (Figure 6A).

### Expression of *CYP82D1.1* and *CYP82D2* in *Arabidopsis*

Flavones are very difficult to detect in *Arabidopsis*, and genes encoding flavone synthase II are absent from its genome (Martens and Mithofer, 2005). Three subfamily genes were found in *Arabidopsis* that encode proteins belonging to the CYP82 subfamily: *CYP82C*, *CYP82G*, and *CYP82F*. CYP82C2 and CYP82C4 can hydroxylate 8-methoxypsoralen to form 5-hydroxy-8-methoxypsoralen (Kruse et al., 2008). CYP82G1 is responsible for the breakdown of (*E,E*)-geranyllinalool to the insect-induced C16-homoterpene (*E,E*)-4,8,12-trimethyltrideca-1,3,7,11-tetraene (TMTT) (Lee et al., 2010). No enzymatic characterization of CYP82F1 in *Arabidopsis* has been reported. No gene encoding a member of the CYP82D subfamily has been found in the *Arabidopsis* genome. Consequently, *Arabidopsis* is an ideal species in which to test the function of flavone hydroxylase genes. To examine whether SbF6H (CYP82D1.1) and SbF8H (CYP82D2) were functional *in planta*, we overexpressed these two genes in transgenic *Arabidopsis* under the control of the CaMV35S promoter, respectively.

**Figure 7:**
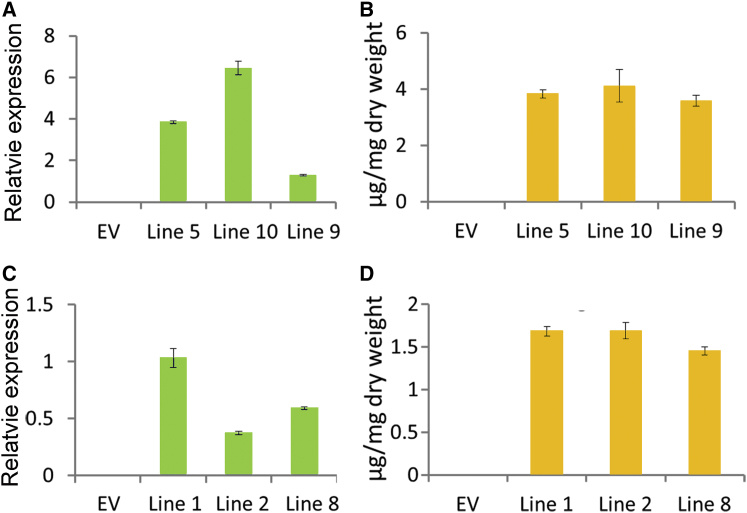
Overexpression of *SbCYP82D1.1* and *SbCYP82D2* in *Arabidopsis*. **(A)** Transcript levels of *SbCYP82D1.1* relative to *Arabidopsis* UBI in an empty vector (EV) control line and three transgenic lines expressing SbCYP82D1.1 under the control of the CaMV35S promoter as determined by qRT–PCR. **(B)** Measurements of baicalein from EV line and three transgenic lines grown on Murashige–Skoog medium supplemented with chrysin. SEs were calculated from three biological replicates. **(C)** Transcript levels of *SbCYP82D2* relative to *Arabidopsis* UBI in EV control line and three transgenic lines determined by qRT–PCR. **(D)** Measurements of norwogonin from empty vector line and three transgenic lines grown on Murashige–Skoog medium supplemented with chrysin. SEs were calculated from three biological replicates for each assay.

Several transgenic lines were obtained and three independent lines were screened for each construct with enhanced expression of target genes (Figure 7A and 7C). T2 seedlings as well as empty vector controls were grown on Murashige–Skoog medium supplemented with chrysin at 50 μM. These plants were extracted and analyzed by LC–MS. Plants expressing SbCYP82D1.1 converted most of the chrysin they absorbed into baicalein, leading to accumulation of baicalein at 3.58–4.12 mg g^−^^1^ DW, while no baicalein was found in empty vector controls (Figure 7B and Supplemental Figure 9). In SbCYP82D2-expressing plants, 8-hydroxylated chrysin was detected in the successful transgenic lines compared with controls, leading to production of 1.45–1.70 mg g^−^^1^ DW norwogonin (Figure 7D and Supplemental Figure 9).

### Reconstitution of the Pathway synthesizing Baicalein in *Nicotiana benthamiana*

**Figure 8:**
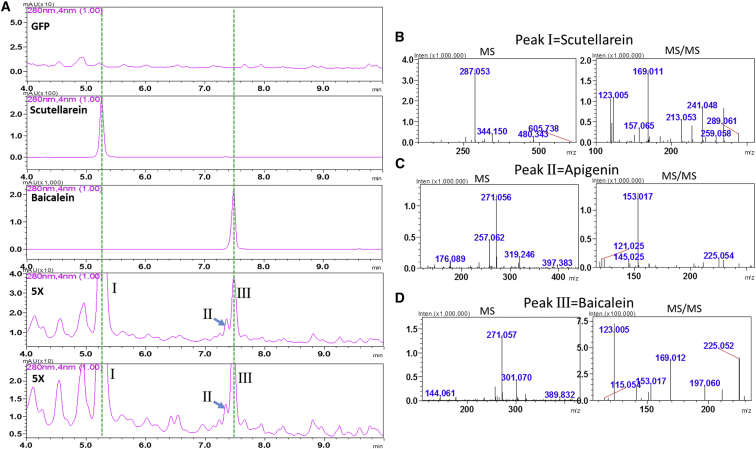
Metabolite Profiles of *N. benthamiana* Leaves Infiltrated with *Agrobacterium tumefaciens* Strain (GV3101) Carrying pEAQ Vectors for Expression of Different Genes. **(A)** Row 1: metabolite profiles of *N. benthamiana* leaf infiltrated with GFP as a control. Row 2: Profile of scutellarein standard. Row 3: baicalein standard. Rows 4 and 5: *N. benthamiana* leaves infiltrated with five *Agrobacterium* strains expressing SbCLL-7, SbCHS-2, CHI, FNSII-2, and CYP82D1 (two replicates). **(B)** MS and MS/MS of peak I from 6X plants, which was identified as scutellarein. **(C)** MS and MS/MS of peak II from 6X plants, which was identified as apigenin. **(D)** MS and MS/MS of peak III from 6X plants, which was identified as baicalein.

*Agrobacterium tumefaciens* strains expressing *SbCLL*-*7*, *SbCHS*-*2*, *SbCHI*, *SbFNSII*-*2*, and *SbCYP82D1.1* in pEAQ vectors were inoculated into leaves of *N. benthamiana* to determine whether together they could reconstitute the entire pathway synthesizing baicalin. As *N. benthamiana* synthesizes apigenin in its leaves, this experiment offered us the opportunity to test whether SbCYP82D1.1 (F6H) could use apigenin as a substrate *in vivo* to produce scutellarein. While there were plenty of flavonoid peaks in the UV spectra of extracts from these inoculated plants, there were clearly three novel peaks not seen in inoculations made with the GFP pEAQ vector alone. One peak, running at just over 7.5 min, ran in the same position as the baicalin standard. The second peak, which ran a little faster, was identified as apigenin. A third peak, not found in extracts inoculated with the GFP vector control, ran at 5.4 min. This was identical to the scutellarein standard. The new peak of scutellarein was present at substantially higher levels than baicalein. MS/MS spectra confirmed the identities of these products (Figure 8).

## Discussion

*S. baicalensis* accumulates large amounts of 4′-deoxyflavones in its roots, which can be used for prescriptions with multiple health-promoting activities. We have shown previously that a newly evolved pathway is involved in the biosynthesis of chrysin, which is then decorated by an F6H to produce baicalein or by an F8H and an 8-OMT (8-*O*-methyltransferase) to make wogonin. Among the five *F6H* candidate genes (*SbCYP71D1*, *SbCYP71D2*, *SbCYP82D1.1*, *SbCYP82D1.2*, and *SbCYP82D2*) analyzed in yeast strains, only SbCYP82D1.1 was able to convert chrysin to baicalein (Figures 2 and 3), showing that it could be the F6H responsible for baicalein biosynthesis.

The first reported CYP82 proteins involved in flavonoid biosynthesis were CYP82D33 from sweet basil and CYP82D62 from mint. These two enzymes have similar functions. They can convert the flavone genkwanin to 7-*O*-methylscutellarein. However, 7-*O*-methylation is a prerequisite for the 6-hydroxylation activity of these two enzymes, as both had very low activity toward apigenin (a flavone without the 7-*O*-methyl group). The SbCYP82D1.1 protein identified from *S. baicalensis* is distinct from CYP82D33 and CYP82D62, and can hydroxylate chrysin efficiently, a flavone containing neither a 7-*O*-methyl group nor a 4′-OH group, to produce baicalein. SbCYP82D1.1 has similar or slightly higher binding affinity for chrysin (*K*_M_ 0.12 μM) than CYP82D33 has for genkwanin (*K*_M_ 0.20 μM). Furthermore, SbCYP82D1.1 exhibits a level of substrate promiscuity, because it can use apigenin, 7-*O*-methylchrysin, and chrysin as substrates with comparable turnover rates (Figure 2), showing that this enzyme could be responsible for synthesis of both baicalein and scutellarein in roots and aerials parts of *S. baicalensis*, respectively. This conclusion is supported by the expression of *SbCYP82D1.1* in both roots and aerial parts. Transcript levels of *SbCYP82D1.1* are comparable in leaf and stem samples and comprise about 40% of the levels found in roots (Figure 3A), which may account for the 5.97 and 8.64 μg mg^−^^1^ DW scutellarein in the leaves and stems of *S. baicalensis*, respectively (Islam et al., 2011). Interestingly, *de novo* synthesis of scutellarein was observed in leaves of *N. benthamiana* inoculated with *Agrobacterium* carrying a construct expressing *CYP82D1.1*, which presumably was synthesized from apigenin (which is made by members of the tobacco family; Jie Luo, personal communication), confirming the broad substrate range of CYP82D2 *in vivo* (Figure 8). However, SbCYP82D1.1 had no activity on the flavanone, pinocembrin, which is distinct from the activity of CYP82D33, since CYP82D33 can convert the flavonone, sakuranetin, at 38.3% of the turnover rate of genkwanin. These data suggest strongly that SbCYP82D1.1 is an enzyme specific for flavones in *S. baicalensis*.

When expression of *SbCYP82D1.1* was knocked down using RNAi in hairy roots, the content of baicalin was reduced significantly, while chrysin glucuronide levels increased (Figure 3D and Supplemental Figure 3). This implied that when flavone 6-hydroxylase was downregulated, its substrate, chrysin, accumulated and was converted by a glycosyl transferase to produce chrysin glucuronide. 7-*O*-Glucosyltransferase activity with broad substrate specificity has been reported in *S. baicalensis* (Hirotani et al., 2000). In CYP82D1.1-silenced lines, chrysin accumulated, but F8H and OMT activities apparently were unaffected. This would explain the enhanced amounts of wogonin and wogonoside in the *SbCYP82D1.1* RNAi lines (Figure 3D) and suggested that the accumulation of different 4′-deoxyflavones is dependent on the relative activities of F6H and F8H, at least in roots of *S. baicalensis*. These findings matched well with our previous study of SbFNSII-2, which uses only pinocembrin as substrate to produce chrysin. Based on the results presented here, we can complete the biosynthetic pathway of baicalein in *S. baicalensis* because the 6-hydroxylase activity of CYP82D1.1 is the final step in the baicalein biosynthetic pathway, following SbFNSII-2 activity.

In addition to CYP82D1.1, there are two other types of 6-hydroxylases involved in flavonoid metabolism. CYP71D9 from soybean 6-hydroxylates flavanones, but this enzyme has very low activity on flavones (Latunde-Dada et al., 2001). We isolated two CYP71D9 homologs from *S. baicalensis*, neither of which, however, had any activity on chrysin, as shown by *in vivo* yeast assays. The third type of flavonoid 6-hydroxylase is an oxoglutarate-dependent dioxygenase from *Crinum americanum* that uses methylated flavonols as substrates. However, no activity of this enzyme on flavones was found (Anzellotti and Ibrahim, 2000, Anzellotti and Ibrahim, 2004). The similar function of the three distinct types of flavonoid 6-hydroxylases suggests strongly that flavonoid 6-hydroxylase activity evolved independently on many occasions as a result of convergent evolution. This may explain why plants, in general, employ different types of enzymes for 6-hydroxylation of different subclasses of flavonoids.

A Rieske-type enzyme was reported to be a flavone 8-hydroxylase in sweet basil, a member of the family Lamiaceae, like *Scutellaria*. Sweet basil ObF8H is responsible for 8-hydroxylation of salvigenin and crisimaritin in peltate trichomes. The homolog of ObF8H from *Scutellaria* (which we called SbRTO: Rieske-type oxygenase) had its highest expression levels in leaves but the lowest in roots (Figure 4B), contrasting strongly with the accumulation patterns of RSFs and with the expression patterns of previously isolated genes involved in RSF biosynthesis, such as *SbCLL*-*7*, *SbCHS*-*2*, and *SbFNSII*-*2*. Our experiments, including a yeast *in vivo* assay and RNAi, ruled out the possibility of SbRTO being the F8H responsible for wogonin and wogonoside synthesis. Furthermore, ObF8H has been reported to be a plastid-localized protein, but the previous biosynthetic step is catalyzed by FNSII-2, which should be an ER-localized CYP450 protein. It would be difficult for a plastid-localized F8H to compete for the common substrate, chrysin, with an ER-localized F6H and to synthesize sizable amounts of wogonin and wogonoside in the roots of *S. baicalensis*. Therefore, *Scutellaria* likely evolved a new F8H activity responsible for norwogonin synthesis, different from the ObF8H from sweet basil. In fact, yeast *in vivo* and *in vitro* enzyme assays demonstrated that SbCYP82D2 encodes an F8H. However, the turnover rate of SbCYP82D2 is quite low compared with SbCYP82D1.1, although SbCYP82D2 is very specific, and can accept only chrysin as substrate among the flavone substrates tested in this study.

Phylogenetic analysis showed that SbCYP82D1.1, SbCYP82D2, and two other CYP82D proteins from salvia cluster together, having diverged from CYP82D33 and CYP82D62. The CYP82D progenitor might have undergone gene duplication in the common ancestor of the family Lamiaceae, establishing two groups of F6H, one of which could efficiently catalyze hydroxylation of only 7-*O*-methylflavones while the other could hydroxylate flavones lacking a 7-*O*-methyl group, such as chrysin. The phylogenetic analysis also suggests that SbCYP82D2 might have evolved from SbCYP82D1.1 following a relatively recent gene duplication event. SbCYP82D2 may have gained its F8H activity via duplication and neofunctionalization, while retaining the ability to 6-hydroxylate as a minor function, from its F6H ancestor. Although SbCYP82D2 is specific for chrysin as substrate, this specificity was achieved at the cost of decreased turnover rate compared with F6H/SbCYP82D1.1. A similar scenario was observed between flavone synthases in the 4′-hydroxy- and 4′-deoxyflavone biosynthetic pathways of *S. baicalensis* (Zhao et al., 2016b). SbFNSII-2/CYP93B25 might have diverged from SbFNSII-1/CYP93B24 after separation of *Scutellaria* from other species of the family Lamiaceae. The newly evolved enzyme SbFNSII-2 is substrate specific, yet rather inefficient, whereas SbFNSII-1 is both an efficient and a promiscuous enzyme (Zhao et al., 2016b). Similar duplication and neofunctionalization of CYP450 enzymes have been described in *Arabidopsis*, where *p*-coumaraldehyde hydroxylase CYP84A4 arose by duplication of the gene encoding the ancestor of CYP84A1, a lignin biosynthetic enzyme (Weng et al., 2012).

Our structural modeling results suggest that several amino acid substitutions occurring in SbCYP82D2 relative to SbCYP82D1.1 may explain differences in their catalytic activities. Bulky residues and a nearby hydrophobic pocket composed of phenylalanine residues in the SbCYP82D1.1 active site may help orient its substrates (chrysin or apigenin) in a tilted conformation in the binding pocket, placing C6 in close proximity (4.9 Å) to the catalytic protoporphyrin iron (Figure 6). In contrast, the lack of bulky residues and nearby phenylalanines in SbCYP82D2 may cause chrysin to bind such that its C8 position becomes closer (4.1 Å) to the protoporphyrin iron of the active site (Figure 6), consistent with its divergent catalytic activity. Results from the silencing of the two CYP450 enzymes suggest that the two enzymes work in competition and that the relative levels of baicalein and wogonin (and their respective glycosides) may be determined, primarily by the relative activities of these two enzymes in roots of *S. baicalensis*. These results are important for designing production systems for different 4′-deoxyflavones, as well as for the 4′-hydroxyflavone scutellarein, which is also sought after for medicinal applications.

## Methods

### Plant Materials and Compounds

Seeds of *S. baicalensis* Georgi were bought from Northern Medicinal Seeds, Anguo county, Hebei province of China. Plants of *S. baicalensis* Georgi were grown in plant chambers at 25°C under a 16-h/8-h photoperiod.

Baicalin, baicalein, scutellarein, wogonin, pinocembrin, chrysin, and apigenin were purchased from Sigma-Aldrich (http://www.sigmaaldrich.com/). Norwogonin was purchased from Extrasynthese (http://www.extrasynthese.com). Wogonoside was purchased from Carbosynthe (http://www.carbosynth.com/). 7-*O*-Methylchrysin was purchased from Yuanye (http://www.shyuanye.com/). The compounds were dissolved in methanol to obtain standard stock solutions (1 mg ml^−^^1^).

### Isolation of the Candidate Genes

The sequences of CYP82D33, CYP71D9, and ObF8H were used for BLAST searches against *Scutellaria* deep-sequencing databases. The ORFs of the genes (*SbCYP82D1.1*, *SbCYP82D1.2*, *SbCYP82D2*, *SbCYP71D1*, *SbCYP71D2*, and *SbRTS*) were isolated using the primers listed in Supplemental Table 1 based on the contigs in the databases and were subcloned into plasmid pDONR207 using Gateway BP Clonase II Enzyme Mix (http://www.thermofisher.com).

Full-length CDSs of *SbCYP82D1.1*, *SbCYP82D1.2*, *SbCYP82D2*, *SbCYP71D1*, and *SbCYP71D2* were cloned into the yeast expression vector pYesdest52. The full-length CDSs of *SbCYP82D1.1* and *SbCYP82D2* were also cloned into plasmid pK7WG2R (Karimi et al., 2002, Ding et al., 2008) for *Arabidopsis* transformation. All constructs were made using Gateway LR Clonase II Enzyme Mix (http://www.thermofisher.com) according to the manufacturer's protocols.

### Yeast *In Vivo* Assays

The yeast expression vector pYesdest52 constructs with *SbCYP82D1.1*, *SbCYP82D1.2*, *SbCYP82D2*, *SbCYP71D1*, *SbCYP71D2*, or an empty vector were transformed into yeast *S. cerevisiae* WAT11 (Truan et al., 1993, Pompon et al., 1996) for expression of the candidate CYP450 proteins. Successful yeast transformants were screened on plates with synthetic dropin medium-Ura (SD-Ura) containing 20 g l^−^^1^ glucose and grown at 28°C for 2 days. Single colonies of the engineered strains were initially grown in 10 ml of SD-Ura liquid medium with 20 g l^−^^1^ glucose at 28°C for about 12 h to an OD_600_ of 2–3. The cells were harvested by centrifugation and resuspended in the SD-Ura supplemented with 20 g l^−^^1^ galactose to induce expression of the target proteins. The substrate, chrysin, was then supplemented at 50 μM into the cultures. After 16 h (for F6H) or 24 h (for F8H), the cells were centrifuged, ice-dried, and extracted with 70% MeOH for LC–MS analysis.

### Enzyme Assays and Kinetics

Recombinant yeast strains were grown as described previously (Zhao et al., 2016b). Target proteins were induced in the SD-Ura liquid medium supplemented with 20 g l^−^^1^ galactose for 16 h. Microsomal proteins were isolated according to the protocol described by Truan et al. (1993) and were dissolved in protein storage buffer (20% [v/v] glycerol, 50 mM Tris–HCl [pH 7.5], and 1 mM EDTA). Protein concentrations were determined using Bradford's assay (Bradford, 1976). The flavone hydroxylases were assayed in 200 μL of reaction volume, which contained 100 mM sodium phosphate buffer (pH 7.9), 0.5 mM reduced glutathione, and 2.5 μg of crude protein extract and 10 μM substrate (chrysin, apigenin, 7-*O*-methylchrysin, or pinocembrin). The assays were initiated by adding NADPH at 1 mM and were incubated for 30 min for F6H or 6 h for F8H at 28°C. MeOH was then added to a final concentration of 70% into the assays to quench the reactions. The samples were filtered through 0.22-μm nylon column and analyzed by LC–MS. Microsomal proteins extracted from yeast harboring the empty vector were assayed as a negative control.

For kinetics measurements, chrysin at varying concentrations from 0.1 to 5 μM was added to the reaction system as above and incubated at 28°C for 3 min for F6H or 30 min for F8H. *K*_M_ and *V*_max_ values were evaluated using an Eadie–Hofstee plot.

### RNAi in Hairy Roots

Non-conserved regions of sequence from *SbCYP82D1.1*, *SbCYP82D2*, and *SbRTO* cDNAs were amplified using primers listed in Supplemental Table 1 and the PCR products were subcloned into pDonr207 using Gateway BP Clonase II Enzyme Mix. The DNA fragments were confirmed by DNA sequencing and were then cloned into RNAi vector pK7WGIGW2R using Gateway LR Clonase II Enzyme Mix. *Agrobacterium rhizogenes* A4 strain was transformed with the RNAi constructs by electroporation. The reconstructed strains were used for inducing hairy roots from leaf explants of *S. baicalensis*.

Young leaf explants collected from *S. baicalensis* plants were firstly treated with 75% methanol for 30 s, then sterilized with 10% bleach for 10 min, and washed with sterile water five times. The explants were then scratched using a knife dipped with *A. rhizogenes* suspension solutions (two to three scratches for each leaf over a central vein), dried on sterile filter-paper, and co-cultured on B5 medium containing 50 μM acetosyringone for 3 days in the dark at 25°C. The explants were then transferred to B5 medium supplemented with 500 mg l^−1^ cefotaxime (Sigma) to remove bacteria. After 2 weeks, hairy roots could be found at the wound site on the explants, and successful transformants were screened for expression of dsRed using a fluorescence microscope and removed from explants. The dsRed-positive hairy roots were kept on B5 medium containing 400 mg/l cefotaxime (Sigma) as separate independent lines at 25°C in the dark.

An elongated hairy root was cut from each line, cultured in a 100-ml flask containing 10 ml of B5 liquid with 400 mg l^−^^1^ cefotaxime, and maintained by shaking (90 rpm) at 25°C. Liquid medium was added gradually up to 50 ml with growth of the roots. After 50 days, the hairy roots were collected and ground in liquid N_2_. The samples were then freeze-dried and extracted in a sonicator with 70% methanol for 2 h. The extracts were filtered using 0.22-μm columns before HPLC analysis.

### Metabolite Analysis

An Agilent 1260 Infinity II HPLC system was used for metabolite analysis. Separation was carried out on a 100 × 2-mm 3μ Luna C18(2) column using 0.1% formic acid in water (A) versus 1:1 ACN/MeOH + 0.1% formic acid (B) and run at 260 μL min^−^^1^ with the following gradient: 0–3 min, 20% B; 20 min, 50% B; 20–30 min, 50% B; 36 min, 30% B; 37 min, 20% B; and 37–43 min, 20% B. The column was maintained at 35°C and absorption was detected at 280 nm with a diode array detector (Agilent). Metabolites were measured by comparing the area of the individual peaks with standard curves obtained from standard compounds.

LC–MS/MS was carried out on an ion-trap TOF mass spectrometer attached to a Prominence/Nexera UHPLC system (Shimadzu). Separation was on a 100 × 2-mm 3μ Luna C18(2) column using the same gradient described above. Flavonoids were detected by UV absorption, collecting spectra from 200 to 600 nm from which we extracted chromatograms at the desired wavelength, 280 nm. They were also detected by positive electrospray MS, collecting spectra from *m*/*z* 200–2000 (using automatic sensitivity control, 70% of base peak chromatogram). The instrument also collected automatic (data-dependent) MS2 spectra of the most abundant precursor ions, at an isolation width of *m*/*z* 3.0, 50% collision energy, and 50% collision gas, with an ion accumulation time of 10 ms. Spray chamber conditions were 250°C curved desorbation line, 300°C heatblock, 1.5 l min^−^^1^ nebulizing gas, and drying gas “on.” The instrument was calibrated using sodium trifluoroacetate before use.

### Phylogenetic Analysis

Protein sequences were aligned using the Clustal X 2 (Jeanmougin et al., 1998). The phylogenetic trees were constructed using maximum-likelihood (ML) methods of MEGA 6 with the following options settings: Poisson substitution model, uniform rates, partial deletion for gaps/missing data, 95% site coverage cutoff, strong branch swamp filter, and 1000 bootstrap replications.

### qRT–PCR

Plant samples were collected and frozen in liquid nitrogen immediately. The samples were ground into fine power and total RNA was extracted from them using RNeasy plant mini kits (Qiagen, http://www.qiagen.com/). First-strand cDNA was synthesized using SuperScript III (Invitrogen, http://www.invitrogen.com/) with oligo(dT)17 primer plus random primers (sigma). Real-time qRT–PCR was performed based on the corresponding cDNA samples using gene-specific primers as shown in Supplemental Table 1 as described previously (Luo et al., 2007).

### Modeling of SbCYP82Ds and Docking Chrysin into Their Active Sites

SbCYP82D1.1 and SbCYP82D2 were modeled using SwissModel, according to the template of zebrafish cytochrome P450 17A2 with Abiraterone (4R20), as this returned the highest sequence identity of all proteins in the RCSB PDB (www.rcsb.org) (Berman et al., 2000, Pallan et al., 2015). The coordinates of the HEM protoporphyrin group were also modeled in SbCYP82D1.1 and SbCYP82D2 according to 4R20. Chrysin from the PDB (57D) was used as the input for docking experiments with AutoDock Vina (Trott and Olson, 2010).

### *Arabidopsis* Transformation

*Arabidopsis thaliana* Columbia-0 was used for transformation. Seeds were sown in soil and stratified at 4°C for 2 days before transfer to a growth chamber. The plants were maintained under long-day (16 h light, 8 h dark) conditions. Temperature and humidity were kept at 23°C and 50%. Plant binary vector pH7WG2R was used for expression of *SbCYP82D1.1* and *SbCYP82D2*, which were driven by CaMV 35S promoter. The constructs as well as an empty vector control were introduced into *Agrobacterium tumefaciens* GV3101 pMP90 by electroporation. The transformed bacteria were used for transformation of *A. thaliana* by *in planta* infiltration (Bent, 2000). Transformed seedlings (T1) were screened on solid Murashige–Skoog medium supplemented with 5 mg l^−^^1^ hygromycin and were then transferred to soil. Positive transgenic plants were confirmed by genomic PCR. Homozygous T3 plants were used in further studies.

Two-week-old sterile *Arabidopsis* seedlings were transferred to 100 ml of Murashige–Skoog solid medium with or without chrysin and cultured under long-day conditions. After 2 weeks, the seedlings were washed with distilled water, freeze-dried, and ground to a fine powder. Samples of 10 mg DW were extracted for metabolites in 1 ml of 70% methanol and then sonicated in an ultrasonic water bath at room temperature for 1 h. The resulting extract was centrifuged at 12 000 *g* for 5 min at 4°C and the supernatant was used for acid hydrolysis. An equal volume of 2 N HCl was added to the samples for incubation at 90°C for 1 h. Filtered samples (10 μL) were used for LC–MS analysis.

### Reconstitution of the 4′-Deoxyflavone Pathway in *N. benthamiana*

Full-length cDNAs of *GFP*, *SbCLL*-*7*, *SbCHS*-*2*, *SbCHI*, *SbFNSII*-*2*, and *SbCYP82D1.1* were cloned into pEAQ-HT-DEST1 and transformed into *A*. *tumefaciens* GV3101 pMP90 (Peyret and Lomonossoff, 2013). Leaves of *N. benthamiana* plants were infiltrated according to the protocol of Geisler et al. (2013). Infiltrated leaves were harvested at 7 days after infiltration and metabolites were extracted and analyzed as described for *Arabidopsis* samples.

### Statistics

All experiments were repeated using at least three biological replicates. Data are presented as means ± SEM, unless stated otherwise. Paired or unpaired, two-tailed Student's *t*-tests were used to compare group differences. *P* values of less than 0.05 were considered significant.

## Funding

This work was supported by National Natural Science Foundation of China (31700268), the Chenshan Special Fund for Shanghai Landscaping Administration Bureau Program G172402 and G162409, and by the CAS/JIC and Center of Excellence for Plant and Microbial Sciences (CEPAMS) joint foundation support to Q.Z., D.Y., X.-Y.C., and C.M. Q.Z., C.M., L.H., and D.Y. were also supported by the Institute Strategic Program Understanding and Exploiting Plant and Microbial Secondary Metabolism (BB/J004596/1) from the BBSRC to JIC. D.Y. was supported by a CSC fellowship from the State Scholarship fund of China. J. Li was supported by a John Innes Foundation Rotation Studentship during the course of this work. This work was also supported by the Pew Scholar Program in the Biomedical Sciences and the Searle Scholars Program to J.-K.W.

## Author Contributions

Q.Z. and C.M. designed and managed the project; Q.Z. isolated the genes and characterized the enzymes; Q.Z., M.-Y.C., and J. Liu performed silencing of genes, gene expression assays, and bioinformatics analysis; O.L. and J.-K.W. modeled the hydroxylases; D.Y. and J. Li reconstructed the pathway in tobacco leaf; L.H. helped in LC–MS analysis; C.M., Q.Z., L.Y., Y.H., J.-K.W., and X.-Y.C. analyzed and interpreted the data; Q.Z. and C.M. wrote the paper with significant input from all authors.

## Accession Numbers

The sequences of the genes isolated in this work were verified by complete gene sequencing and have been submitted to the NCBI database with the accession numbers: *SbCYP82D1.1*, MF363006; *SbCYP82D1.2*, MF363007; *SbCYP82D2*, MF363008; *SbCYP71D1*, MF363004; *SbCYP71D2*, MF363005; and *SbRTO*, MF363009.
