# Supplementary material for: Two CYP82D Enzymes Function as Flavone Hydroxylases in the Biosynthesis of Root-Specific 4′-Deoxyflavones in Scutellaria baicalensis
Source: Mol Plant. 2018 Jan 8;11(1):135–48. doi: 10.1016/j.molp.2017.08.009 (PMC5770198; doi:10.1016/j.molp.2017.08.009)
Supplement: Document S1. Supplemental Figures 1–10 and Supplemental Tables 1–3 [file mmc1.pdf]

Supplementary Table 1. Structures of Compounds related to this work

Compounds related to this work	Structures
baicalin	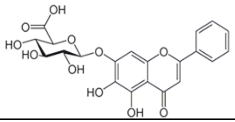
baicalein	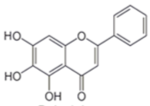
wogonoside	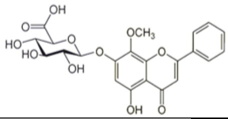
wogonin	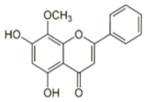
scutellarin	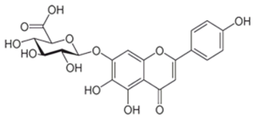
scutellarein	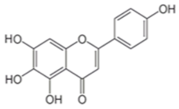
pinocembrin	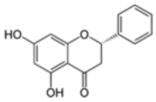
chrysin	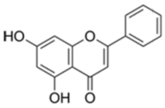
norwogonin	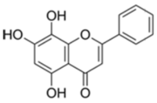
naringenin	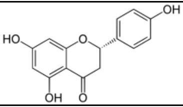
apigenin	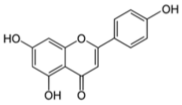

Supplementary Table 2. FPKM of candidate flavone hydroxylase genes

	**Gene ID**	**Expression (FPKM) HairyRoots**	**Expression (FPKM) Flowers**
SbCYP82D1	CL5673.Contig4_All	59.7978	10.7494
SbCYP82D2	CL5510.Contig2_All	195.0871	3.3727
SbCYP71D1	CL3280.Contig1_All	3.464	1.9377
SbCYP71D2	Unigene16719_All	127.4283	8.5701

Supplementary Table 3. Primers used in this study

Names	Used for	Sequences
SbCYP82D1 CDS F	Cloning	GGGGACAAGTTTGTACAAAAAAGCAGGCTTCATGGAGTTGAGCTCTGTCATCTATG
SbCYP82D1 CDS R	Cloning	GGGGACCACTTTGTACAAGAAAGCTGGGTTTTAATATAGAGTAGGTGACAACCTTGG
SbCYP82D2 CDS F	Cloning	GGGGACAAGTTTGTACAAAAAAGCAGGCTTCATGCTATATGACAACAGCCAATAC
SbCYP82D2 CDS R	Cloning	GGGGACCACTTTGTACAAGAAAGCTGGGTTCTAGTAAAGAGTGGGAGACAAC
SbCYP71D1 CDS F	Cloning	GGGGACAAGTTTGTACAAAAAAGCAGGCTTCATGGACATCCAATTTCCCTCACTC
SbCYP71D1 CDS R	Cloning	GGGGACCACTTTGTACAAGAAAGCTGGGTATCATGGCAAAGGTCTTGTAATAATAAGAG
SbCYP71D2 CDS F	Cloning	GGGGACAAGTTTGTACAAAAAAGCAGGCTTCATGGGGTTCCTCATTCTCCTCTC
SbCYP71D2 CDS R	Cloning	GGGGACCACTTTGTACAAGAAAGCTGGGTACTTCTAGTCCCTCTCTCTTTGCAAC
SbRTO CDS F	Cloning	GGGGACAAGTTTGTACAAAAAAGCAGGCTTCATGGAAGCTCTCAAAGCTTCATC
SbRTO CDS R	Cloning	GGGGACCACTTTGTACAAGAAAGCTGGGTTTCAACGGAAGGCATGGTCATAG
SbCYP82D1.1 QPCR F	qPCR	CTACGACGGAGCCTCGCTTG
SbCYP82D1.1 QPCR R	qPCR	TGGCTCGCTGCAGCTCGATC
SbCYP82D2 QPCR F	qPCR	ATTGGACGGAATTGTTGGA
SbCYP82D2 QPCR R	qPCR	TCACTTCCACCCACAATCAA
SbCYP71D1 QPCR F	qPCR	GTGAATTGCACGAAGAAGCA
SbCYP71D1 QPCR R	qPCR	GTCCACTGTTGTGGATGACG
SbCYP71D2 QPCR F	qPCR	CGACACCATTCTTGAGAGCA
SbCYP71D2 QPCR R	qPCR	CTCGGTTTCTTCACCAGCTC
SbRTO QPCR F	qPCR	CCAGAGAGTGTGAAGGCTGA
SbRTO QPCR R	qPCR	TTTGGTTGCACTTGAGGACG
SbActin QPCR F	qPCR	TCTTGATCTTGCTGGTCGTG
SbActin QPCR R	qPCR	CACTGCAGAGCTGGTCTTTG
SbCYP82D1 RNAi F	RNAi	GGGGACAAGTTTGTACAAAAAAGCAGGCTTCACATGAAGCAGTGGCTGGGGAAC
SbCYP82D1 RNAi R	RNAi	GGGGACCACTTTGTACAAGAAAGCTGGGTTTGATGGTATCAGCATCGTAGTGGAG
SbCYP82D2 RNAi F	RNAi	GGGGACAAGTTTGTACAAAAAAGCAGGCTTCATCTCATGGCTGGTGGCACCAC
SbCYP82D2 RNAi R	RNAi	GGGGACCACTTTGTACAAGAAAGCTGGGTTCACACTGTCCGAACCCTCACTTC
SbRTO RNAi F	RNAi	GGGGACAAGTTTGTACAAAAAAGCAGGCTTCATGGAAGCTCTCAAAGCTTCATC
SbRTO RNAi R	RNAi	GGGGACCACTTTGTACAAGAAAGCTGGGTTCACCAACCATGGTACACACAC

Underlined sequences represent Gateway® primers

Supplementary Figure 1. HPLC profile of *in vitro* enzyme assay of CYP82D1.1 in yeast

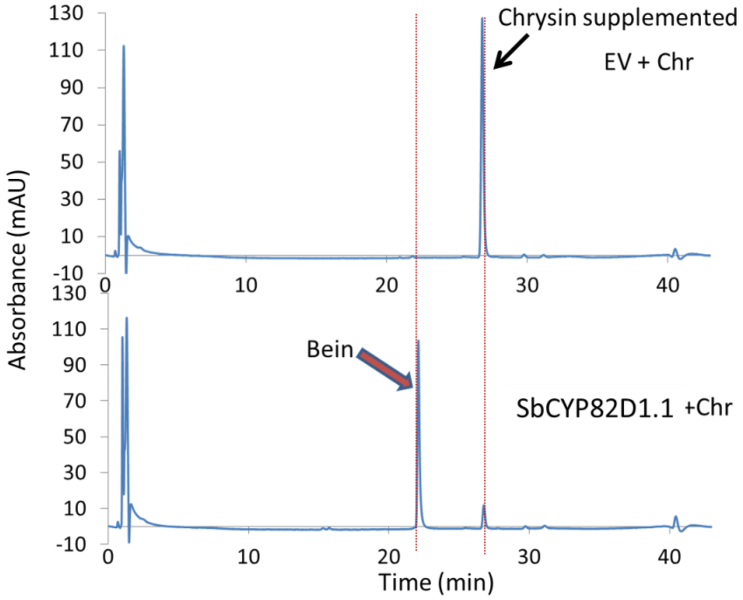

EV = Empty Vector; Chr = Chrysin; SbCYP82D1.1 = yeast expressing SbCYP82D1.1

Supplementary Figure 2. HPLC profiles of *in vitro* enzyme assays of CYP82D1.1 supplied with different substrates

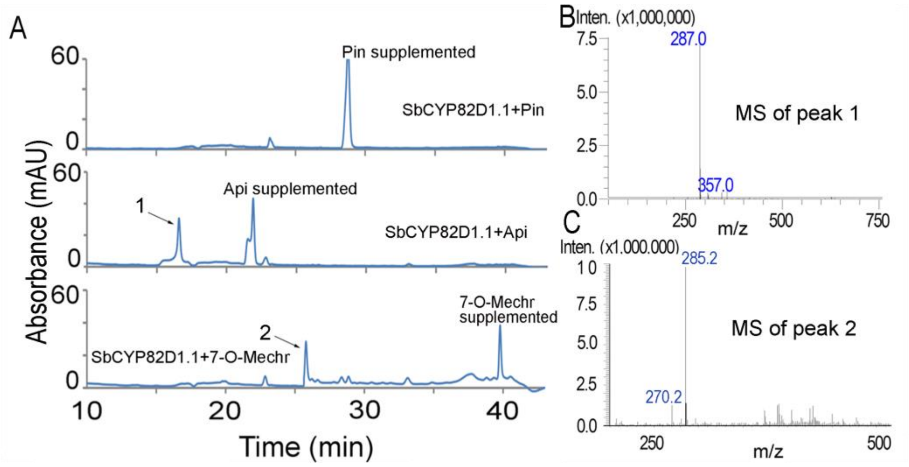

Supplementary Figure 2. *In vitro* enzyme assays of CYP82D1.1 supplied with different substrates.

(A) HPLC analysis of CYP82D1.1 with: Top; pinocembrin (Pin), Middle; apigenin (Api) and Bottom; 7-O-methylchrysin supplied as substrates. Peak 1 (scutellarein) and Peak 2 (negletein) were detected from reactions with apigenin and 7-O-methylchrysin, respectively. **(B)** MS of Peak 1 confirming this to be scutellarein and **(C)** Peak 2 confirming this to be negletein.

Supplementary Figure 3. LC-MS analysis of flavonoids in *SbCYP82D1.1* RNAi hairy roots

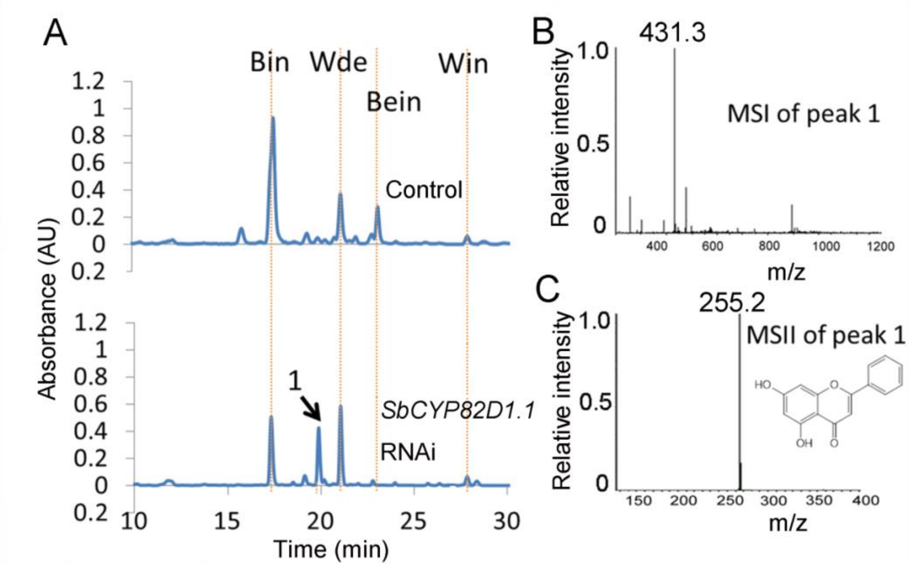

Supplementary Figure 3. LC-MS analysis of flavonoids in *SbCYP82D1.1* RNAi hairy roots

(A) HPLC analysis of empty vector control and *SbCYP82D1.1* RNAi hairy roots. Peak 1 was detected only in *SbCYP82D1.1* RNAi hairy roots. **(B)** MSI of Peak 1 and **(C)** MSII of Peak 1 showing this to be chrysin glucuronide. Bin = Baicalin; Bein = Baicalein; Win = Wogonin; Wde = Wogoniside.

Supplementary Figure 4. ***In vivo*** **assay of SbRTO with chrysin in yeast**

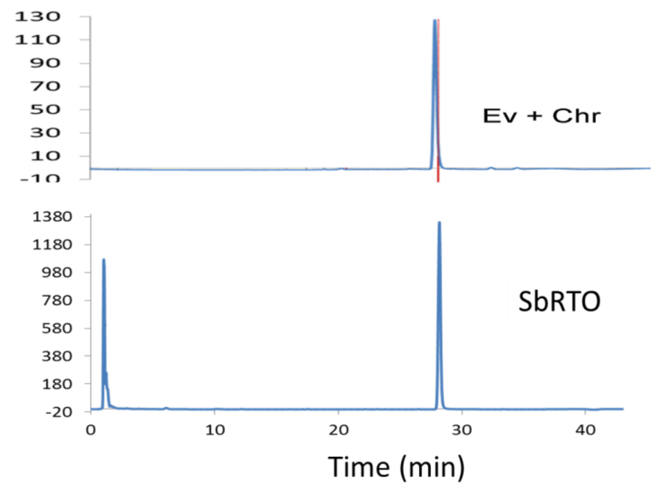

Supplementary Figure 4. ***In vivo*** **assay of SbRTO with chrysin in yeast**

No new products were observed following feeding of yeast expressing SbRTO with chrysin, showing that SbRTO can not use chrysin as a substrate. Ev = Empty vector; Chr = chrysin

Supplementary Figure 5. ***In vitro*** **enzyme assay of CYP82D2 expressed in yeast**

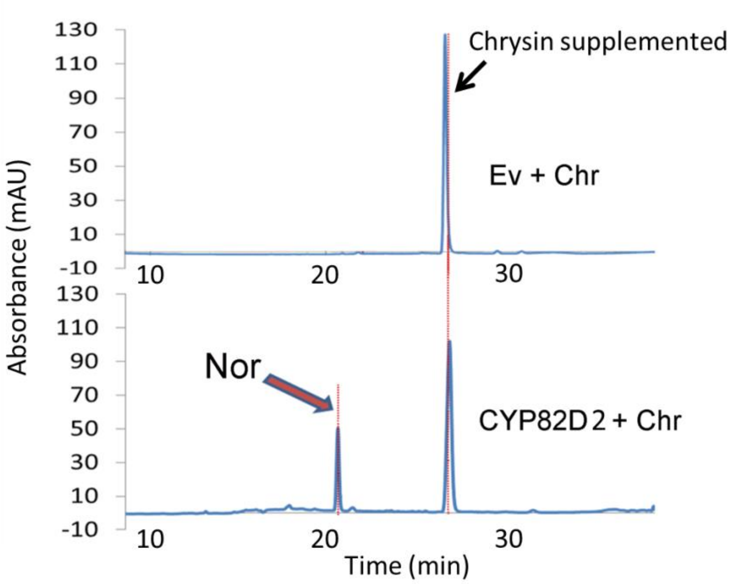

Supplementary Figure 5. ***In vitro*** **enzyme assay of CYP82D2 expressed in yeast**

Microsomes from yeast expressing SbCYP82D2 and supplied with chrysin (Chr) formed a new peak of norwogonin (Nor) compared to microsomes from empty vector control (Ev) yeast.

Supplementary Figure 6. *In vitro* enzyme assays of CYP82D2 with different substrates

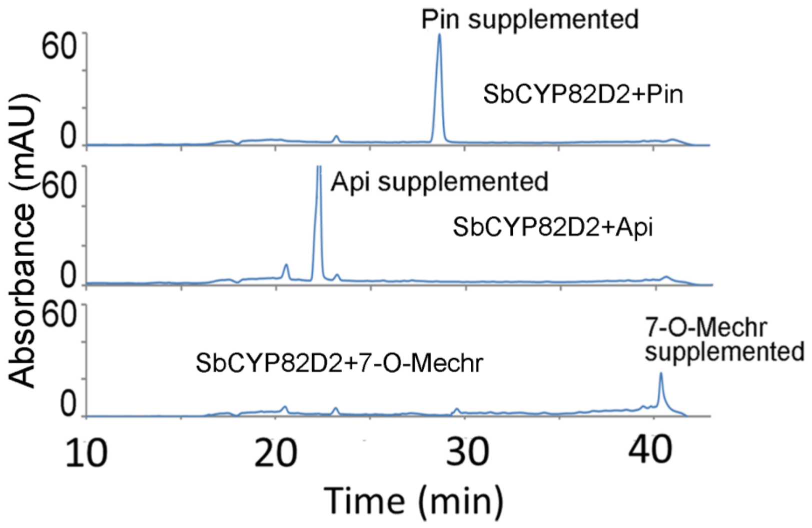

Supplementary Figure 6. *In vitro* enzyme assays of CYP82D2 with different substrates

Microsomes prepared from yeast expressing SbCYP82D2 failed to make any new products when supplied with Pinocembrin (Pin), Apigenin (Api) or 7-Methylchrysin (7-O-Mechr).

Supplementary Figure 7. HPLC analysis of flavonoids in *SbCYP82D2* RNAi hairy roots

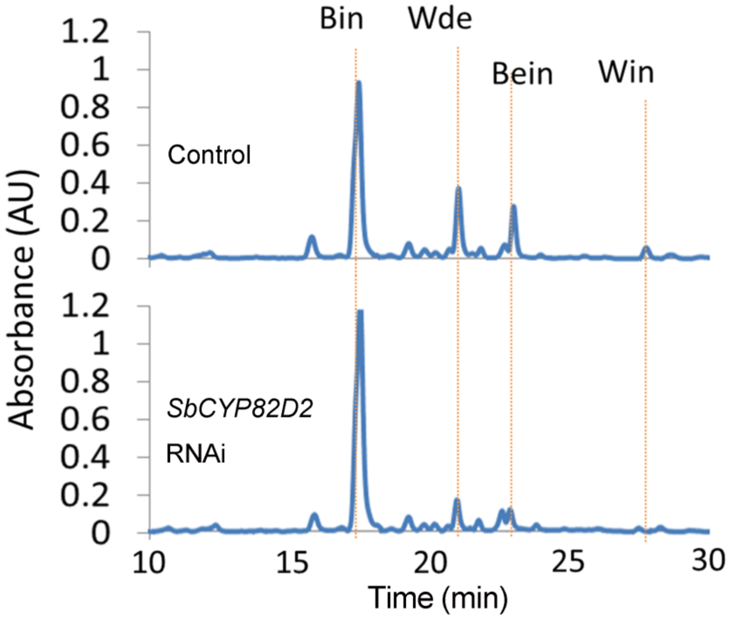

Supplementary Figure 7. HPLC analysis of flavonoids in *SbCYP82D2* RNAi hairy roots

Levels of Baicalein (Bein), Baicalin (Bin), Wogonin (Win) and Wogoniside (Wde) in hairy roots silenced for SbCYP82D2, compared to an empty vector control.

Supplementary Figure 8. SbCYP82D2 can also produce baicalein in small amounts

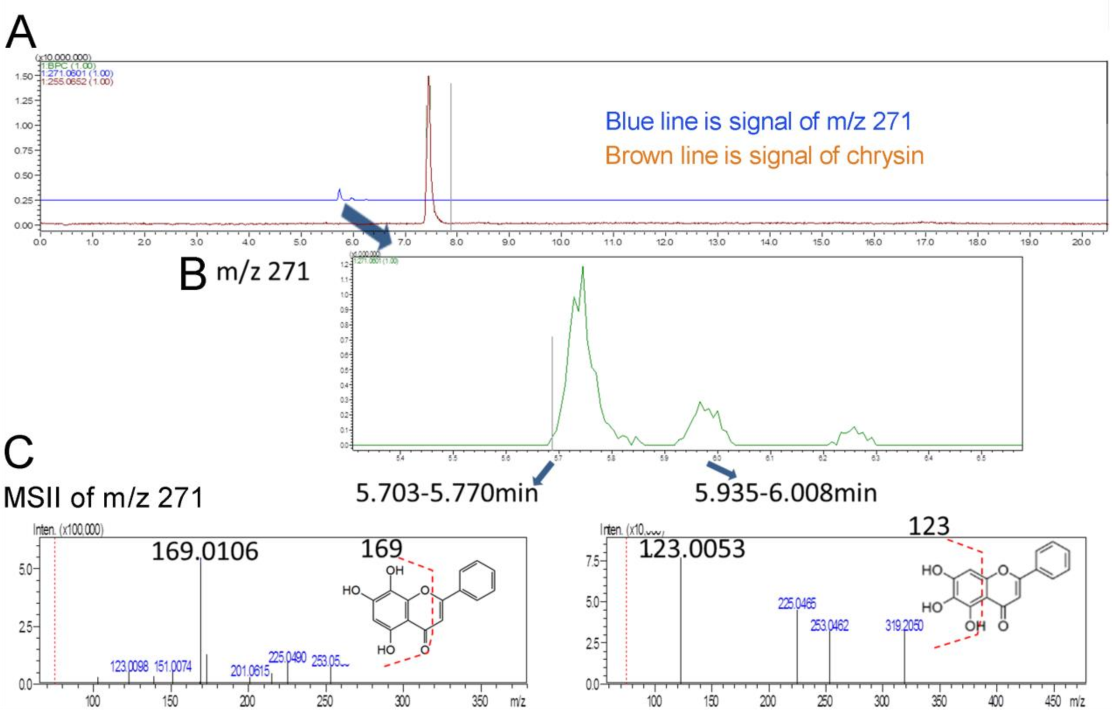

Supplementary Figure 8. SbCYP82D2 can also produce baicalein in small amounts

(A) HPLC trace of flavonoids made following *in vivo* assay of CYP82D2 expressed in yeast and fed chrysin. Blue line shows new products compared to brown line showing chrysin peak
(B) Higher resolution profile of new peaks of m/z I of 271
(C) MSII of first peak with m/z 271 showing this to be norwogonin, and of second peak showing this to be baicalein

Supplementary Figure 9. HPLC analysis of flavonoids in transgenic Arabidopsis expressing either SbCYP82D1.1 or SbCYP82D2 and fed chrysin

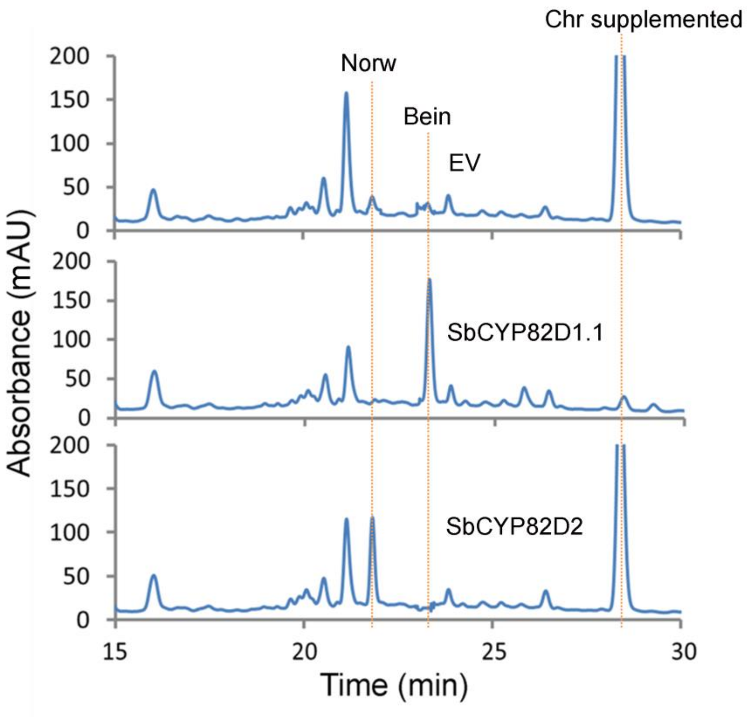

Supplementary Figure 9. HPLC analysis of flavonoids in transgenic Arabidopsis expressing either SbCYP82D1.1 or SbCYP82D2 and fed chrysin

Norw = Norwogonin; Bein = Baicalein; EV = Empty Vector; Chr = Chrysin

Supplementary Figure 10. Multiple amino acid sequence alignments for phylogentic trees.

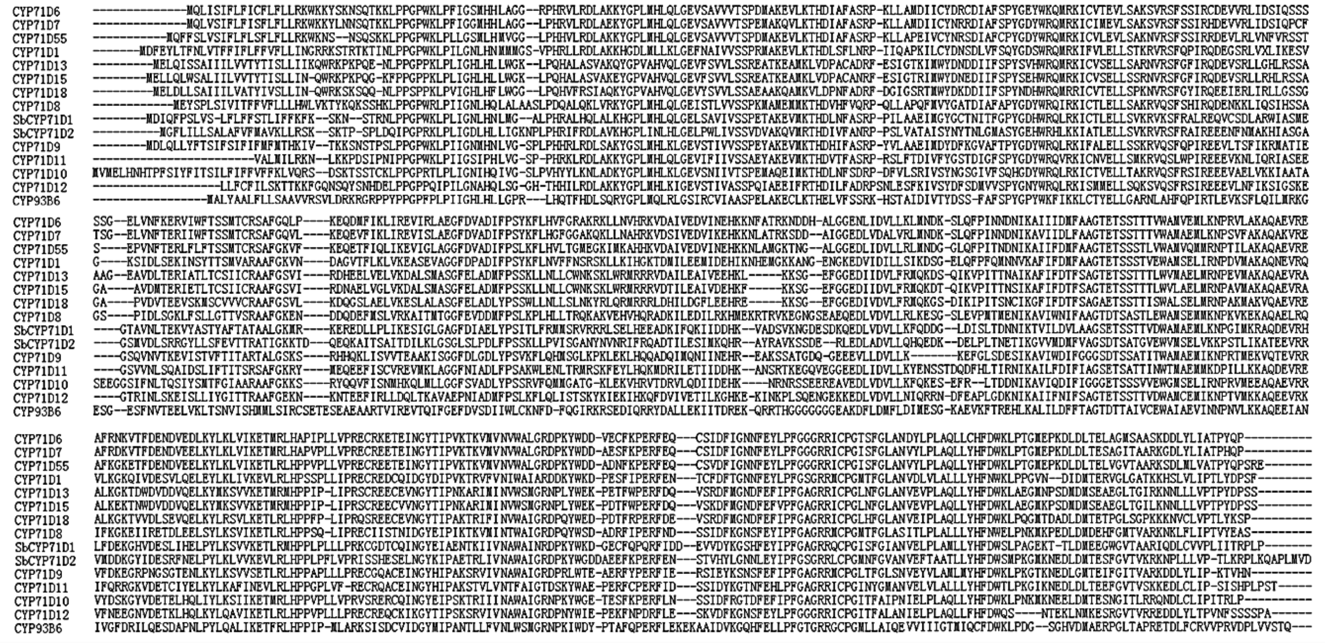

Multiple alignment of CYP71D proteins

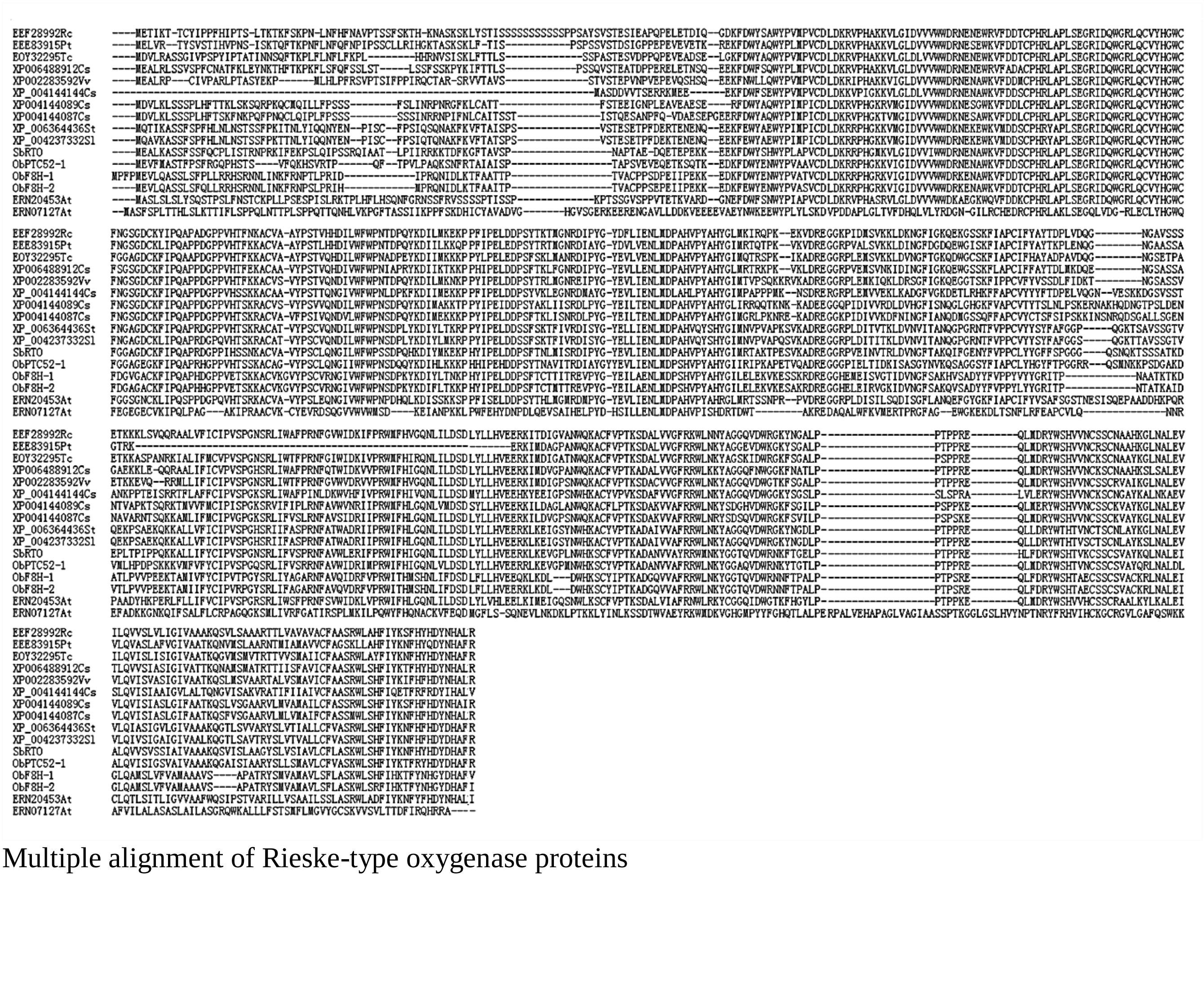

Multiple alignment of Rieske-type oxygenase proteins

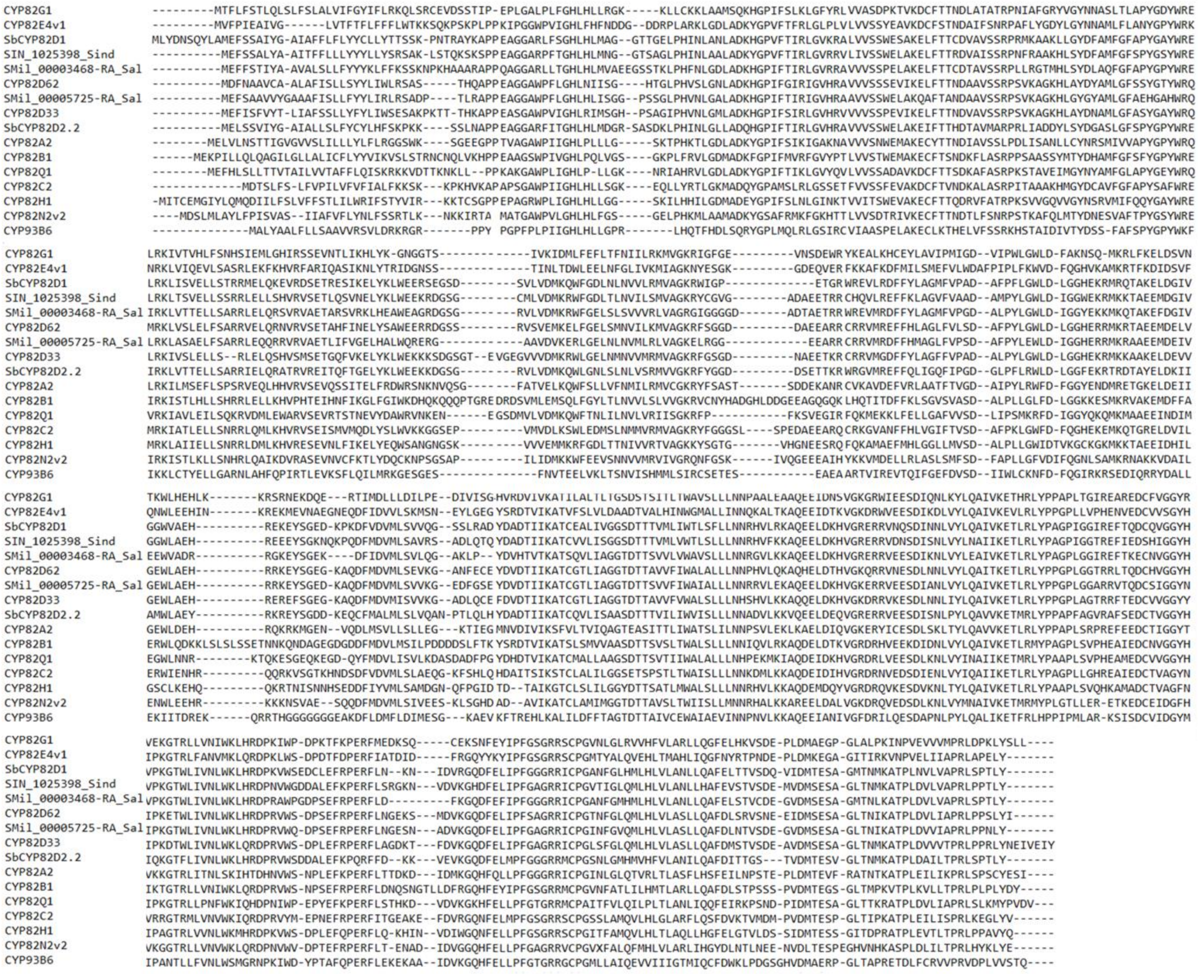

Multiple alignment of CYP82D proteins
